# Supplementary material for: Error correcting optical mapping data
Source: Gigascience. 2018 May 25;7(6):giy061. doi: 10.1093/gigascience/giy061 (PMC6007263; doi:10.1093/gigascience/giy061)
Supplement: GIGA-D-17-00139_Original_Submission.pdf [file giy061_giga-d-17-00139_original_submission.pdf]

GigaScience
Error Correcting Optical Mapping Data
--Manuscript Draft--

**Manuscript Number:**	GIGA-D-17-00139
**Full Title:**	Error Correcting Optical Mapping Data
**Article Type:**	Research
**Funding Information:**	Division of Information and Intelligent Systems (1618814) — Dr. Christina Boucher
**Abstract:**	Optical mapping is a unique system that is capable of producing high-resolution, high-throughput genomic map data that gives information about the structure of a genome (Schwartz et al., Science 1993). Recently it has been used for scaffolding contigs and assembly validation for large-scale sequencing projects, including the maize (Zhou et al., PLoS Genetics, 2009), goat (Dong et al., Nature Biotech. 2013), and amborella (Chamala et al., Science 2013) genomes. However, a major impediment in the use of this data is the variety and quantity of errors in the raw optical mapping data, which are called Rmaps. The challenges associated with using Rmap data—and thus, optical mapping data—is analogous to dealing with insertions and deletions in the alignment of long reads. Moreover, they are arguably harder to tackle since the data is integral and susceptible to inaccuracy. We develop cOMet to error correct Rmap data, which to the best of our nowledge is the only optical mapping error correction ethod. Our experimental results demonstrate that cOMet orrects 82.49% of insertion errors and 77.38% of deletion errors in Rmap data generated from the E. coli K-12 reference genome. It also successfully scales to large genomes , improving the quality of 78% and 99% of the Rmaps in the plum and goat genomes, respectively.
**Corresponding Author:**	Kingshuk Mukherjee<br>University of Florida<br>Gainesville, Florida UNITED STATES
**Corresponding Author Secondary Information:**	
**Corresponding Author's Institution:**	University of Florida
**Corresponding Author's Secondary Institution:**	
**First Author:**	Kingshuk Mukherjee
**First Author Secondary Information:**	
**Order of Authors:**	Kingshuk Mukherjee<br>Darshan Washimkar<br>Martin Muggli<br>Leena Salmela<br>Christina Boucher
**Order of Authors Secondary Information:**	
**Opposed Reviewers:**	
**Additional Information:**	
**Question**	**Response**
Are you submitting this manuscript to a special series or article collection?	No
**Experimental design and statistics**	Yes

Powered by Editorial Manager® and ProduXion Manager® from Aries Systems Corporation

Full details of the experimental design and statistical methods used should be given in the Methods section, as detailed in our Minimum Standards Reporting Checklist. Information essential to interpreting the data presented should be made available in the figure legends.<br><br>Have you included all the information requested in your manuscript?	
**Resources**<br><br>A description of all resources used, including antibodies, cell lines, animals and software tools, with enough information to allow them to be uniquely identified, should be included in the Methods section. Authors are strongly encouraged to cite Research Resource Identifiers (RRIDs) for antibodies, model organisms and tools, where possible.<br><br>Have you included the information requested as detailed in our Minimum Standards Reporting Checklist?	Yes
**Availability of data and materials**<br><br>All datasets and code on which the conclusions of the paper rely must be either included in your submission or deposited in publicly available repositories (where available and ethically appropriate), referencing such data using a unique identifier in the references and in the "Availability of Data and Materials" section of your manuscript.<br><br>Have you have met the above requirement as detailed in our Minimum Standards Reporting Checklist?	Yes

Main tex file Click here to download Manuscript main.tex

OXFORD (GIGA)^n SCIENCE

GigaScience, 2017, 1–7

doi: xx.xxxx/xxxx
Manuscript in Preparation
Paper

PAPER

Error Correcting Optical Mapping Data

Kingshuk Mukherjee[1,*], Darshan Washimkar[2], Martin D. Muggli[2], Leena Salmela[3] and Christina Boucher[1,*]

[1]Department of Computer and Information Science and Engineering, University of Florida, Gainesville and [2]Department of Computer Science, Colorado State University, Fort Collins and [3]Department of Computer Science, Helsinki Institute for Information Technology HIIT, University of Helsinki

[*]kingdgp@ufl.edu; cboucher@cise.ufl.edu

Abstract

Optical mapping is a unique system that is capable of producing high-resolution, high-throughput genomic map data that gives information about the structure of a genome (Schwartz et al., Science 1993). Recently it has been used for scaffolding contigs and assembly validation for large-scale sequencing projects, including the maize (Zhou et al., PLoS Genetics, 2009), goat (Dong et al., Nature Biotech. 2013), and amborella (Chamala et al., Science 2013) genomes. However, a major impediment in the use of this data is the variety and quantity of errors in the raw optical mapping data, which are called Rmaps. The challenges associated with using Rmap data—and thus, optical mapping data—is analogous to dealing with insertions and deletions in the alignment of long reads. Moreover, they are arguably harder to tackle since the data is integral and susceptible to inaccuracy. We develop cOMet to error correct Rmap data, which to the best of our knowledge is the only optical mapping error correction method. Our experimental results demonstrate that cOMet corrects 82.49% of insertion errors and 77.38% of deletion errors in Rmap data generated from the *E. coli* K-12 reference genome. It also successfully scales to large genomes , improving the quality of 78% and 99% of the Rmaps in the plum and goat genomes, respectively.

Key words: Optical mapping; Error correction.

Introduction

In 1993 Schwartz et al. developed *optical mapping*, a system for creating an ordered, genome-wide, high-resolution restriction map of a given organism's genome. Since this initial development, genome-wide optical maps have found numerous applications including discovering structural variations and rearrangements [18], scaffolding and validating contigs for several large sequencing projects [5, 7, 3], and detecting misassembled regions in draft genomes [12]. Thus, optical mapping has assisted in the assembly of a variety of species–including various prokaryote species [14, 23, 24], rice [25], maize [26], mouse [4], goat [5], parrot [7], and *amborella trichopoda* [3]. The raw optical mapping data is generated by a biological experiment in which large DNA molecules cling to the surface of a microscope slide using electrostatic charge and are digested with one or more restriction enzymes. The restriction enzymes cut the DNA molecule at occurrences of the enzyme's recognition sequence, forming a number of DNA fragments. The fragments formed by digestion are painted with a fluorescent dye, to allow visibility under laser light and a CCD camera.Computer vision algorithms then estimate fragment length from consolidated intensity of fluorescent dye and apparent distance between fragment ends.

The resulting data from an experiment are in the form of an ordered series of fragment lengths [27]. The data for each single molecule produced by the system is called an *Rmap*. Rmap data has a number of errors due to the experimental conditions and system limitations. In an optical mapping experiment, it is unlikely to achieve perfectly uniform fluorescent staining. This leads to an erroneous estimation of fragment sizes. Also, restriction enzymes often fail to digest all occurrences of their recognition sequence across the DNA molecule. This manifests as missing restriction sites. Additionally, due to the fragile na-

Compiled on: June 21, 2017.
Draft manuscript prepared by the author.

ture of DNA, additional breaks can incorrectly appear as restriction sites. Lastly, the limitations of the imaging component of the optical mapping system and the propensity for the DNA to ball up at the ends introduces more sizing error for smaller fragments. Because of all these experimental conditions, Rmap data generated through optical mapping experiment has insertions and deletions of cut sites along with fragment size substitution errors.

Nonetheless, in order to use Rmap data for further analysis. the Rmaps have to be assembled into a genome wide optical map. This is because the single molecule maps need redundant sampling to overcome the presence of the aforementioned errors, and because single molecule maps only span on the order of 500 Kbp [19]. The first step of this assembly process involves finding pairwise alignments amongst the Rmaps. In order to accomplish this, the challenge of dealing with missing fragment sizes has to be overcome. This challenge is analogous to dealing with insertions and deletions in the alignment of long reads—in fact, it is arguably harder since the data is numerical. At present, the only non-proprietary algorithmic method for pairwise alignment of Rmaps is the dynamic programming based method of Valouev et al. [19].

This method is inherently computationally intensive but if the error rate of the data could be improved then non-dynamic-programming based methods that are orders of magnitude faster such as Twin [11], OMBlast [8], and Maligner [10] could be used for alignment. This would greatly improve the time required to assemble Rmap data. Thus, we present cOMet in order to address this need. To the best of our knowledge, it is the first Rmap error correction method. Our experimental results demonstrate that cOMet corrects 82.49% of insertion errors and 77.38% of deletion errors in Rmap data generated from the *E.coli* K-12 reference genome. It also successfully scales to large genomes, improving the quality of 78% and 99% of the Rmaps in the plum and goat genome, respectively.

Background

From a computer science perspective, optical mapping can be seen as a process that takes in two strings: a nucleotide sequence $S_i[1, n]$ and a restriction sequence $B[1, b]$, and produces an array (string) of integers $R_i[1, m]$. The array R_i is an Rmap corresponding to S_i and contains the string-lengths between cuts produced by B on S_i. Formally, R_i is defined as follows: $R_i[j] = y - x$ where y represents the location(starting index) of jth occurrence of B in S_i and x represents the location of $(j-1)^{th}$ occurrence of B in S_i and $R_i[1] = y - 1$ and $R_i[m] = n - x$. For example, say we have $B = act$ and $S_i = atacttactggactactaaact$. The locations of B in S_i are as follows: 3,7,12,15,20. Then R_i will be represented as $R_i = 2, 4, 5, 3, 5, 2$.

We note that millions of Rmaps are produced for a single genome since optical mapping is performed on many cells of the organism and each cell provides thousands of Rmaps. The Rmaps can be assembled to produce a genome wide optical map. This is analogous to next generation shotgun sequencing where Rmaps are analogous to reads and a genome-wide optical map is analogous to the assembled whole genome.

There are three types of errors that can occur in optical mapping: (1) missing cut sites which are caused by an enzyme not cleaving at a specific site, (2) additional cut sites which can occur due to random DNA breakage and (3) inaccuracy in the fragment size due to the inability of the system to accurately estimate the fragment size. Continuing again with the example above, a more representative example Rmap would include these errors, such as $R'_i = 7, 6, 3, 4$. There is a 15% probability that a cut site is missing, i.e., error type (1) occurs in Rmap. For every 400 Kbp of DNA, there is about one random break appearing as a cut site, i.e. error type (2). The inaccuracy of the fragment sizes, i.e., error type (3), follows a normal distribution with standard deviation (σ) which depends on actual length of the fragment. For example [20], if L is the actual length of a DNA fragment on the microscope slide, then the length measured by the optical mapping system shows a normal distribution across L with variance

$$\sigma^2 = f(L). \tag{1}$$

In practice, when aligning a pair of Rmaps, one should allow for twice the error rate of a single Rmap since each Rmap will deviate from the genomic map by the above parameters.

Valouev et al. [19] provides a dynamic programming algorithm for pairwise alignment, which generates a score for every possible alignment between two Rmaps and returns the alignment which achieves the highest score, which is referred to as the S-score. It is computed within a standard dynamic programming framework, similar to Smith and Waterman alignment [17]. The scoring function is based on a probabilistic model built on the following assumptions: the fragment sizes follow an exponential distribution, the restriction sites follow an independent Bernoulli process, the number of false cuts in a given genomic length is a Poisson process, and the sizing error follows a normal distribution with mean zero and variance following a linear function of the true size. Lastly, a different sizing error function is used for fragments less than 4 kbp in length since they do not converge to the defined normal distribution. The score of an alignment is calculated as the sum of two functions; one function that estimates and scores the sizing error, and a second that predicts and scores the presence of additional and/or missing cut sites between the fragments. The S-score will be used later in this paper to evaluate the error correction process.

Methods

Given a set of n Rmaps $\mathrm{R} = \{R_1, .., R_n\}$ our method aims to detect and correct all errors in R by considering each $R_i \in \mathrm{R}$ and finding a set of Rmaps that originate from the same part of the genome as R_i. This step is performed heuristically in order to avoid aligning every pair of Rmaps in R.

Preprocessing

Our first step is to remove the first and last fragments from each Rmap in R. These fragments have one of their edges sheared by artifacts of the DNA prep process (preceding the optical mapping process) and not by restriction enzymes. Unless removed, they can misguide alignment between two Rmaps during the error correction process. In addition, short Rmaps, i.e., those that have less than 10 fragments, are removed at this stage since any Rmap that contains less than 10 fragments is typically deemed too small for analysis even in consensus maps [1]. Next, the data is quantized so a given genomic fragment will often be represented by the same value across multiple Rmaps despite the noise. Our quantization method assigns a unique value to a range of fragment sizes by dividing each fragment size by a fixed integer, denoted as b, and rounding to the nearest integer. The quantized data is used to find the set of *related* Rmaps as explained in the next section.

Finding Related Rmaps

We refer to two Rmaps as *related* if their corresponding error-free Rmaps originate from overlapping regions of the genome.

	1st	2nd	3rd	4th	5th	6th	7th		8th	9th	10th	11th	12th	13th	
R_i	1.474	3.625	2.092	2.164	8.424	2.331	24.824		7.267	2.954	12.578	2.358	8.955	22.943	
R_j		3.331	4.464		8.287	2.481	10.314	13.391	7.711	3.143	8.448	5.921	13.795	4.143	6.119

A_i	1st	2nd	3rd	4th	5th	6th	7th	8th	9th	10th	11th	12th	13th
:	:	:	:	:	:	:	:	:	:	:	:	:	:
j	(0,0)	(1,1)	(2,1)	(2,1)	(1,1)	(1,1)	(1,2)	(1,1)	(1,1)	(2,2)	(2,2)	(2,3)	(2,3)
:	:	:	:	:	:	:	:	:	:	:	:	:	:

Figure 1. An alignment between R_i and R_j as given by Valouev et al. [19] and its corresponding entry in the multiple alignment grid A_i. Each column of A_i represents one fragment from R_i and each row represents one Rmap from its' set of related Rmaps. The fragment sizes are in Kbp.

Next, we define a k-mer as a string of k consecutive fragments from a (quantized) Rmap. For example if we have the Rmap $R = \{3, 3, 5, 2, 6, 5, 5, 1\}$ and k=4 then the following k-mers can be extracted from R: (3,3,5,2), (3,5,2,6), (5,2,6,5), (2,6,5,5) and (6,5,5,1). In order to avoid aligning all pairs of Rmaps to find the related Rmaps, we use the number of common k-mers to discriminate between pairs of Rmaps that are related and those that are not. To accomplish this efficiently, we first extract all unique k-mers in each quantized Rmap, and construct a hash table storing each unique k-mer as a key and the list of Rmaps containing an occurrence of that k-mer as the value. Next, we consider each R_i in R and use this hash table to construct the set of Rmaps that have m or more k-mers in common with R_i. Unfortunately, this set, although it contains all related Rmaps, it also likely contains Rmaps that are not related to R_i. Therefore, we filter this set of Rmaps using a heuristic that tries to match each Rmap in this set with R_i in order to ascertain if it is related to R_i. The heuristic traverses through two Rmaps(R_i and one Rmap from the set, say R_j) attempting to match subsets of the fragments from each until it either reaches the end of one Rmap or it fails to match the fragments. We start the traversal from the first matching k-mer between R_i and R_j. We denote the position of the next fragment to be matched in R_i and R_j as x and y, respectively, and assume that each fragment prior to these positions is matched. Next, we consider all combinations of matching the fragments at positions x, $x + 1$ and $x + 2$ of R_i with fragments at positions y, $y + 1$ and $y + 2$ of R_j, and select the combination whose difference in the total size is least; if there exists a tie, we select the match that has the least number of added or missing cut sites. If this selected match leads to a difference in size that is greater than a specified threshold (which was set to 25% of the larger sized fragment in practice), then we conclude that there is not a match at these positions and return that R_i and R_j are unrelated. Otherwise, we increment x and y accordingly and move onto the next fragments. If this heuristic continues until the last fragment of either R_i or R_j is reached then we return that R_i and R_j are related. Using this heuristic we filter out the Rmaps that were deemed to be related based on the number of k-mers in common with R_i but are infact unrelated to R_i.

Rmap Alignment

Next, for each R_i in R, we use the alignment method of Valouev et al. [19] to find the S-score of all pairwise alignments between R_i and each Rmap in its set of related rmaps. The Rmaps that have an alignment score, i.e., S-score less than a defined threshold (which was set to 8 in practice), are removed from the set of related Rmaps and the alignments of the remaining Rmaps are stored in a *multiple alignment grid*, denoted as A_i. This grid is a two-dimensional array of integer pairs, where the number of rows is equal to the number of remaining Rmaps in the set of related Rmaps of R_i and the number of columns is equal to the number of fragments in R_i. An element of this array, $A_i[j, k]$ stores an integer pair in the form of (x, y) representing that x fragments of R_i, (which includes the k-th fragment of R_i) matches to y fragments of R_j in the optimal alignment between R_i and R_j. Figure 1 illustrates an example of A_i. The first fragment of R_i does not match with any fragment of R_j and therefore, $(0, 0)$ is stored at this position. Fragments 2, 5, 6, 8 and 9 of R_i each matches with one fragment of R_j, e.g., 1, 3, 4, 7 and 8, respectively. To represent these matches, we store a (1,1) in 2nd, 5th, 6th, 8th and 9th column of row j. Fragments 3 and 4 of R_i match with one fragment of R_j, i.e., the 2nd fragment. To represent this, we store (2,1) in $A_i[j, 3]$ and $A_i[j, 4]$. Fragment 7 of R_i matches with two fragments of R_j, i.e., the 5th and 6th fragments. To represent this, we store (1,2) in $A_i[j, 7]$. Fragments 10 and 11 of R_i match with two fragments of R_j, i.e., the 9th and 10th fragments. To represent this, we store (2,2) in positions $A_i[j, 10]$ and $A_i[j, 11]$. Finally, fragments 12 and 13 match with three fragments of R_j, i.e., fragments 11, 12 and 13. In this case, we store (2,3) in positions $A_i[j, 12]$ and $A_i[j, 13]$.

Error Correcting Using the Consensus

The multiple alignment grid is used to find the consensus grid, denoted as C_i, for Rmap R_i. The grid C_i is a one-dimensional array of integer pairs with size equal to the number of fragments in R_i. The grid is constructed for each R_i in R by iterating through each column of A_i and finding the most frequent integer pair, breaking ties arbitrarily. The most frequent integer-pair is stored at each position of C_i if the frequency is above a given threshold d; otherwise, (0,0) is stored. Figure 2 illustrates the construction of a consensus grid from an alignment grid. The type of error in each fragment of R_i can be identified using $C_i[k] = (x, y)$ as follows: if x and y are equal then a sizing error occurs at the k-th fragment of R_i, otherwise, if x is greater than y then an additional cut site exists, and lastly, if x is less than y then a missing cut site exists. Next, we use C_i and A_i to correct these errors in R_i. For each fragment of R_i, we consider the consensus stored at the corresponding position of C_i, identify the positions in the corresponding column of A_i that are equal to it, and replace the fragment of R_i with the mean total fragment size computed using the values at those positions in A_i. If C_i is equal to (0,0) at any position then the

Multiple alignment grid (A_i)

A_i	1st	2nd	3rd	4th	5th	6th	7th	8th	9th	10th	11th	12th	13th
1	(1,1)	(2,2)	(2,2)	(1,1)	(2,1)	(2,1)	(1,2)	(1,1)	(1,1)	(2,2)	(2,2)	(1,1)	(1,2)
2	(0,0)	(1,1)	(2,1)	(2,1)	(1,1)	(1,1)	(1,2)	(1,1)	(1,1)	(2,2)	(2,2)	(2,3)	(2,3)
3	(0,0)	(1,1)	(2,1)	(2,1)	(1,1)	(1,1)	(1,2)	(1,1)	(1,1)	(1,1)	(1,1)	(1,1)	(1,2)
4	(0,0)	(1,1)	(2,1)	(2,1)	(1,1)	(1,1)	(1,2)	(1,1)	(1,1)	(2,2)	(2,2)	(2,3)	(2,3)
5	(1,1)	(1,1)	(2,1)	(2,1)	(2,1)	(2,1)	(3,3)	(3,3)	(3,3)	(1,1)	(1,1)	(1,1)	(1,2)
6	(0,0)	(1,1)	(2,1)	(2,1)	(1,1)	(1,1)	(1,2)	(1,1)	(1,1)	(2,2)	(2,2)	(2,3)	(2,3)

Consensus grid (C_i)

C_i	(0,0)	(1,1)	(2,1)	(2,1)	(1,1)	(1,1)	(1,2)	(1,1)	(1,1)	(2,2)	(2,2)	(1,1)	(1,2)

Error Correction

<table>
<tr><td>R_i</td><td>1.474</td><td>3.625</td><td>2.092</td><td>2.164</td><td>8.424</td><td>2.331</td><td colspan="2">24.824</td><td>7.267</td><td>2.954</td><td>12.578</td><td>2.358</td><td>8.955</td><td colspan="2">22.943</td></tr>
<tr><td>C_i</td><td>(0,0)</td><td>(1,1)</td><td>(2,1)</td><td>(2,1)</td><td>(1,1)</td><td>(1,1)</td><td colspan="2">(1,2)</td><td>(1,1)</td><td>(1,1)</td><td>(2,2)</td><td>(2,2)</td><td>(1,1)</td><td colspan="2">(1,2)</td></tr>
<tr><td>R'_i</td><td>1.474</td><td>3.273</td><td colspan="2">4.421</td><td>8.754</td><td>2.608</td><td>9.988</td><td>13.891</td><td>7.184</td><td>3.472</td><td>8.532</td><td>6.032</td><td>10.038</td><td>5.633</td><td>15.869</td></tr>
</table>

Figure 2. Example of Multiple alignment grid and consensus grid. The figure shows the multiple alignment grid A_i for an Rmap R_i and its consensus grid C_i. The figure also demonstrates error correction using the consensus grid, with the error corrected Rmap denoted as R'_i. The fragment sizes are in Kbp.

fragment at that position in R_i remains unchanged since it implies that there is no definitive result about the type of error in that position. In addition, if consecutive positions in C_i are discordant then the fragments in those positions in R_i also remains unchanged. For example, if there is a (2,1) consensus at some position of C_i, then we expect the preceding or successive position to also have a (2,1) consensus. However, if this is not the case, then we do not error correct those fragments since the consensus is discordant at those positions. Figure 2 shows this error correction. As it is illustrated, to error correct the second fragment of R_i, we compute the average of the matched fragments from related Rmaps 2, 3, 4, 5 and 6 and replace the second fragment of R_i with that value as shown in Figure 2. Similarly, to correct the third fragment in this example, we identify that (2,1) is in the consensus, which implies that majority of the related Rmaps are such that two fragments of R_i match with one fragment from the set of related Rmaps, and therefore, replace the third and fourth fragments with the average from the corresponding Rmaps and positions.

Datasets

We perform experiments on both simulated and real data. For the real data, we used the Rmap data from the plum [22] and domestic goat [5] sequencing projects. The genome size and number of Rmaps for these species are shown in Table 1. In addition, we simulated Rmap data from *E. coli* K-12 substr. MG 1655 as follows: first, the reference genome was copied 300 times and ten uniformly distributed random loci were selected for each of these copies. These loci form the ends of single molecule that would undergo *in silico* digestion. Next, molecules smaller than 250 Kbp were discarded and the cleavage sites for the RsrII enzyme were then identified within each of these simulated molecules. This error free Rmap data is used for validating the output of our method. Lastly, deletion, insertion and sizing errors were incorporated into the error-free Rmaps. Deletion and insertion errors were simulated by removing a cut site randomly for every 6.66 cut sites, and randomly adding an extra cut site for every 400 kbp, respectively. Finally, the sizing errors were added to each fragment size by first computing the standard deviation using Equation 1 and then sampling from an appropriately parametrized Gaussian distribution. This method of simulating Rmaps was based on the error model described earlier. This simulation resulted in 2,505 Rmaps, containing 7,485 deletion and 554 insertion errors.

Table 1. Summary of the real and simulated data. Rmaps with less than 10 fragments were omitted from all the experiments. cOMet was ran on the remaining 2,504, 548,779 and 3,049,439 Rmaps for the *E. coli*, plum and goat genomes, respectively.

Genome	Size	No. of Rmaps
E. coli	4.6 Mbp	2,504
Plum	284 Mbp	749,895
Goat	2.66 Gbp	3,447,997

Experiments and Discussion

All experiments were performed on Intel E5-2698v3 processors with 192 GB of RAM running 64-bit Linux. The input parameters to cOMet include: b (quantization bucket size), k (k-mer value), m (the number of k-mers needed to be conserved between two Rmaps) and d (the minimum number of Rmaps required to form consensus at a position). The default parameters are b=4000, k=4, m=1 and d=3, and led to the best result across all datasets.

Experiments with Simulated Data

The cOMet error correction was ran on the simulated *E. coli* data. The corrected Rmaps were then aligned to the error-free Rmaps to determine the number of corrected insertions and deletions. The results of this experiment are shown in Table 2. To determine the quality of error correction, we computed the true positive rate (TPR), which is the ratio between the number of insertion (or deletion) errors that cOMet correctly identified and removed and the number of insertion (deletion) errors, and the false positive rate (FPR), which is the ratio between the number of insertion (or deletion) errors that cOMet incorrectly identified and removed, and the total number of fragments not containing an insertion (deletion) error. The TPR is 82.49% and 77.38% with respect to the number of corrected insertions

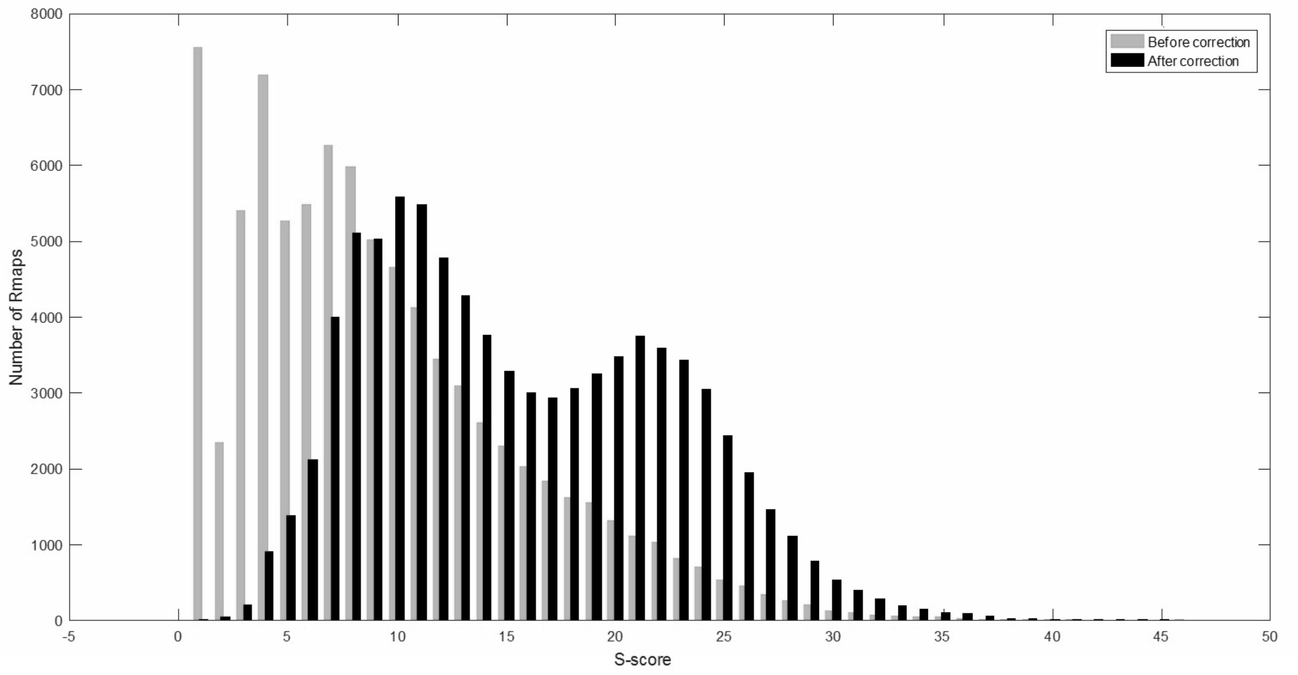

Figure 3. Alignment scores of Rmaps from plum genome with the reference optical-map. Before error correction, the S-score had a mean of 8.6 with standard deviation 6.49. After error correction, the mean S-score improved to 14.72, with standard deviation 6.72.

and deletion errors; whereas, the FPR is 0.21% and 0.25% with respect to the number of corrected insertions and deletion errors. This demonstrates the high accuracy of the correction made by cOMet.

Table 2. Results on the data simulated from *E.coli* K-12 MG 1655. The data was simulated according the algorithm described in Datasets. This simulation resulted in 2,505 Rmaps, containing 7,485 deletion and 554 insertion errors.

Total no. of insertion errors corrected	556
TPR of corrected insertions	82.49 % (457)
FPR of corrected insertions	0.21 % (99)
Total no. of deletion errors corrected	5,894
TPR of corrected deletions	77.38 % (5,792)
FPR of corrected deletions	0.25 % (102)

Additionally, for each corrected Rmap we computed the alignment S-score of both the original Rmap and the corrected Rmap with the error-free Rmap. We found that for 96.5% of the Rmaps, the S-scores improved after error correction. In other words, cOMet brought 96.5% Rmaps closer to their error-free state. The mean S-score before error-correction was 44.91 and it improved by 14.03% to 51.30 after error correction. For 17.5% of the Rmaps, (415 Rmaps) the S-score improved by more than ten. Lastly, we mention that the error correction was achieved in 241 CPU seconds and using 79.54 MB of memory.

Experiments with Real Data

Table 3 summaries the results of running cOMet on the plum and goat datasets. The plum and goat datasets do not contain error-free Rmaps. Therefore, we are restricted to reporting the number of corrections made and the improvement to the S-score. In order to compute the S-score before and after error correction, we generated an in silico digested genome-wide optical map from the reference genome and aligned both the uncorrected and corrected Rmap to the genome-wide optical map. If it aligned to multiple positions then we considered the alignment position where the corrected Rmap aligned with greatest S-score, and considered the difference in the S-score when the uncorrected and corrected Rmap aligned to that position. However, we note that this process is error prone because of the fragmented nature of the draft genomes and possible misassemblies present in the genomes. We observed that the S-score after error correction improved for 78% of the plum Rmaps and 99% of the goat Rmaps. Figures 3 and 4 show the histograms of the distribution of S-scores, before and after error correction. For the plum genome, the mean S-score improved from 8.60 before error correction, to 14.72 after error correction (a 71% improvement in the score) while for the goat genome, it improved from 9.38 before correction to 16.97 after correction (a 80.92% improvement in the score).

Table 3. Results on the Rmap data of plum and goat genomes. Peak memory was measured as the maximum resident set size as reported by the operating system with sufficient RAM to avoid paging. Running time is the user process time, also reported by the operating system.

**Genome name**	**Plum**	**Goat**
Running time	7.4 days	105.7 days
Memory	12.20 GB	113.56 GB
No. of insertion errors corrected	433,282	2,530,060
No. of deletion errors corrected	430,329	3,187,023

We also measured the *genome coverage*, i.e. the fraction of the genome covered by at least one Rmap, for both the original Rmaps and the corrected Rmaps as follows. First we aligned all Rmaps to the genome-wide optical map and then picked the best alignment for each original Rmap and each corrected Rmap. Based on these alignments we then computed the fraction of the genome covered by at least one original Rmap and the fraction of the genome covered by at least one corrected Rmap. On the goat genome the genome coverage was 73.08% before correction and it increased to 84.56% after correction.

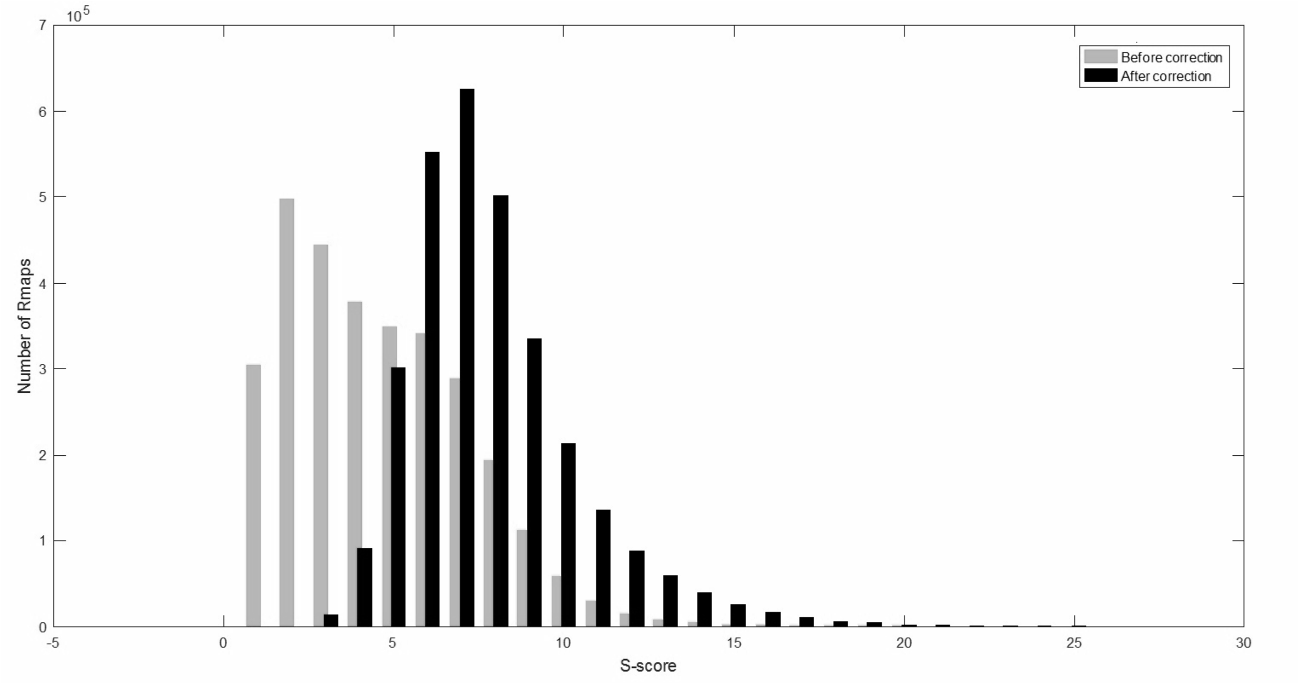

Figure 4. Alignment scores of rmaps from goat genome with the reference optical-map. The mean and standard deviation of the S-scores before error correction were 9.38 and 6.54, respectively. After error correction, the mean S-score improved to 16.97 with a standard deviation of 6.21.

The increase in genome coverage shows that our method is able to correct Rmaps from across the genome. Furthermore it shows that even if Rmaps could not originally be reliably aligned to some regions of the genome, our method is sensitive enough to recover similar Rmaps from these regions and thus after correction the fraction of the genome covered by aligned Rmaps is higher. For the plum, the genome coverage dropped negligibly from 99.01% before error correction to 98.85% after error correction.

In addition, as shown in Table 3, the running time and peak memory usage was recorded for the plum and goat genome. Although these experiments have significant running times, (7.4 and 105.7 CPU days for plum and goat, respectively) these figures are not prohibitive given that this computation can easily be parallelized since the error correction process for each Rmap is independent. For example, we ran the goat genome on 20 machines and thus, it required a total of 126.84 hours for all Rmaps to be corrected. In addition, we note that error correction of a dataset will likely only be done for once for any dataset so 5.2 human days for a large genome is not unreasonable. Lastly, the peak memory usage was 12.20 GB and 113.56 GB, for plum and goat, respectively, and thus, cOMet is able to run on any modern server.

Conclusion

Error correction of high-throughput sequencing data has become an imperative pre-processing step in genome assembly since 2008 when Chaisson and Pevzner showed the dramatic improvement it can have on the quality of the assembly [2, 6, 16]. For example, after error correction the contig N50 size of an assembly of *Rhodabacter sphaeroides* improved from 233 bp to 7,793 bp using the same assembler [16]. Due to this inarguable benefit on genome assembly, countless methods have been developed for error correction of sequence reads, including BFC [9], Coral [15], EULER [13, 2] and Reptile [21]. Unfortunately, even though there has been a massive effort into error correction of sequence data, there currently does not exist a publicly released method for error correction of Rmap data—a method that would likely improve the quality of genome-wide optical map assemblies, and allow such assemblies to be computed with greater efficiency.

In this paper, we presented cOMet, an error correction method for Rmap data, and demonstrate that it corrects and improves the quality of a high percentage of Rmaps in both the simulated and real datasets. As previously discussed, Rmap data is subject to high error rates. In addition to insertion and deletion errors, they contain sizing errors which necessitates the use of dynamic programming algorithm for pairwise alignment, and subsequently, assembly. By correcting a significant number of errors in Rmap data, cOMet can make it possible to use faster alignment methods [11, 8, 10], and explore the development of more efficient Rmap assembly algorithms.

Availability of source code

The cOMet software is written in C++ and is publicly available under GNU General Public License at https://github.com/kingufl/cOMet

Availability of Supporting Data

The optical mapping data for plum and goat is publicly available and can be accessed from their respective manuscripts. The simulated data for *E.coli* is provided in the github repository along with the python scripts used to generate it.

Acknowledgements

KM, DW, MM and CB were funded by the National Science Foundation (1618814) and LS was funded by Academy of Finland (grant 284598 (CoECGR)).

References

1. Bradnam KR, et al. Assemblathon 2: Evaluating *de novo* methods of genome assembly in three vertebrate species. GigaScience 2013;2(1):1–31.
2. Chaisson MJ, Brinza D, Pevzner PA. De novo fragment assembly with short mate-paired reads: Does the read length matter? Genome Res 2009;19(2):336–346.
3. Chamala S, et al. Assembly and Validation of the Genome of the Nonmodel Basal Angiosperm *Amborella*. Science 2013;342(6165):1516–1517.
4. Church DM, et al. Lineage-Specific Biology Revealed by a Finished Genome Assembly of the Mouse. PLoS Biology 2009;7(5):e1000112+.
5. Dong Y, et al., Sequencing and automated whole-genome optical mapping of the genome of a domestic goat (*Capra hircus*). Nature Biotechnol.; 2013.
6. Ekblom R, Wolf JBW. A field guide to whole-genome sequencing, assembly and annotation. Evolutionary Applications 2014;7(9):1026–1042.
7. Ganapathy G, et al. *De novo* high-coverage sequencing and annotated assemblies of the budgerigar genome. GigaScience 2014;3:11.
8. Leung AKY, et al. OMBlast: alignment tool for optical mapping using a seed-and-extend approach. Bioinformatics 2016;p. btw620.
9. Li H. BFC: correcting Illumina sequencing errors. Bioinformatics 2015;31(17):2885.
10. Mendelowitz LM, et al. Maligner: a fast ordered restriction map aligner. Bioinformatics 2016;32(7):1016–1022.
11. Muggli MD, Puglisi SJ, Boucher C. In: Efficient indexed alignment of contigs to optical maps; 2014. p. 68–81.
12. Muggli MD, Puglisi SJ, Ronen R, Boucher C. Misassembly detection using paired-end sequence reads and optical mapping data. Bioinformatics 2015;31(12):i80–i88.
13. Pevzner PA, Tang H, Waterman MS. An Eulerian Path Approach to DNA Fragment Assembly. Proceedings of the National Academy of Sciences 2001;98(17):9748–9753.
14. Reslewic S, et al. Whole-Genome Shotgun Optical Mapping of *Rhodospirillum Rubrum*. Appl Environ Microbiol 2005;71(9):5511–5522.
15. Salmela L, Schröder J. Correcting errors in short reads by multiple alignments. Bioinformatics 2011;27(11):1455–1461.
16. Salzberg SL, et al. GAGE: A critical evaluation of genome assemblies and assembly algorithms. Genome Res 2012;22(3):557–567.
17. Smith TF, Waterman MS. Identification of common molecular subsequences. J Mol Biol 1981;147(1):195 – 197.
18. Teague B, et al. High-Resolution Human Genome Structure by Single-Molecule Analysis. Proc Natl Acad Sci USA 2010;107(24):10848–10853.
19. Valouev A, et al. Alignment of optical maps. J Comp Biol 2006;13(2):442–462.
20. VanSteenHouse H; 2013. personal communication.
21. Yang X, Dorman KS, Aluru S. Reptile: representative tiling for short read error correction. Bioinformatics 2010;26(20):2526.
22. Zhang Q, et al., Genomic data of the plum (Prunus mume). GigaScience Database; 2014.
23. Zhou S, et al. A Whole-Genome Shotgun Optical Map of *Yersinia pestis* Strain KIM. Appl Environ Microbiol 2002;68(12):6321–6331.
24. Zhou S, et al. Shotgun Optical Mapping of the Entire *Leishmania major* Friedlin Genome. Mol Biochem Parasitol 2004;138(1):97–106.
25. Zhou S, et al. Validation of Rice Genome Sequence by Optical Mapping. BMC Genomics 2007;8(1):278.
26. Zhou S, et al. A Single Molecule Scaffold for the Maize Genome. PLoS Genetics 2009 11;5:e1000711.
27. Zhou S, Herschleb J, Schwartz DC. A single molecule system for whole genome analysis. Perspectives in Bioanalysis 2007;2:265–300.

logo file Click here to download Figure example-grid-100x100pt.png

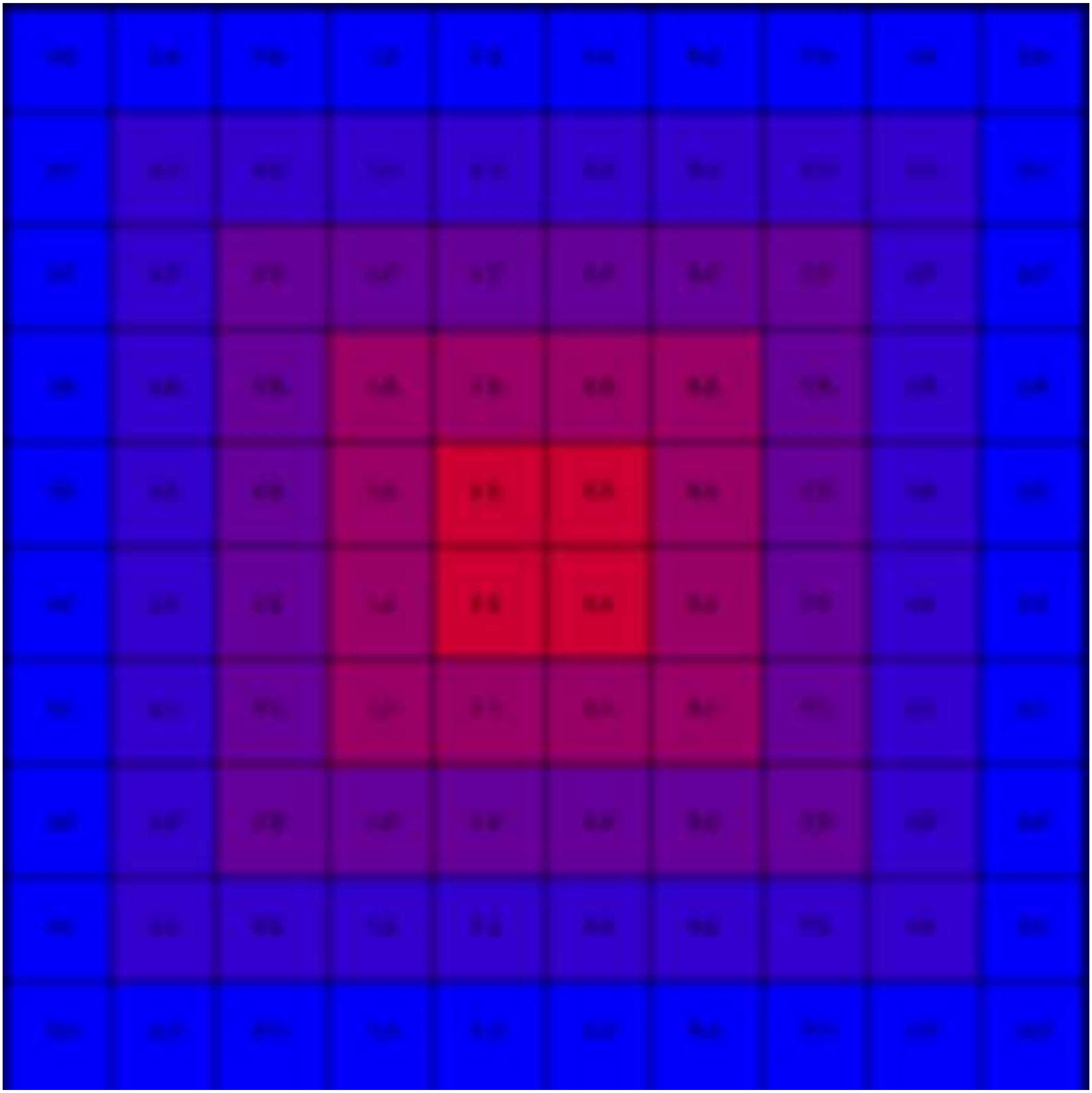

logo file

Click here to download Figure gigascience-logo.eps

logo file

Click here to download Figure oup.eps

OXFORD

Reference pdf

Click here to access/download
Supplementary Material
error-correcting-optical.pdf

UF UNIVERSITY of FLORIDA

Herbert Wertheim College of Engineering
Department of Computer and Information Science and Engineering

300 Weil Hall
PO Box 116550
Gainesville, FL 32611-6550
352-392-6000
352-392-9673 Fax
christinaboucherl@ufl.edu

Dr. Laurie Goodman
Editor-in-Chief
GigaScience

May 30, 2017

Dear Dr. Goodman:

I am pleased to submit an original research article entitled "Error Correcting Optical Mapping Data" by Kingshuk Mukherjee, Darshan Washimker, Martin Muggli, Leena Salmela, and myself. This manuscript builds on our prior work on succinct data structures, error correction and optical mapping.

In this manuscript, we develop Comet, which is the first method proposed and released that error corrects Rmap data. Our experimental results demonstrate that it corrects 82.49% of insertion errors and 77.38% of deletion errors in Rmap data generated from the E. coli K-12 reference genome. It also successfully scales to large genomes, improving the quality of 78% and 99% of the Rmaps in the Plum and Goat genomes, respectively. We suggest Dr. David Schwartz as an editor. Further, we believe that this manuscript is appropriate for publication by GigaScience because of the algorithmic scope and focus on optical mapping, for which the suggested editor is a renowned expert.

This manuscript has not been published and is not under consideration for publication elsewhere. We have no conflicts of interest to disclose. In addition, if you feel that the manuscript is appropriate for your journal, we suggest the following reviewers:

Dr. Ali Bashir
Assistant Professor
Department of Genetics and Genomic Sciences
Icahn School of Medicine at Mount Sinai
EMAIL: ali.bashir@mssm.edu
PHONE: (212) 824-8949

Dr. Chan Ting Fung
Professor
School of Life Sciences
The Chinese University of Hong Kong
EMAIL: tf.chan@cuhk.edu.hk
PHONE (852) 3943 1216

Dr. Anton Valouev
Assistant Professor
Department of Preventive Medicine
Keck School of Medicine
University of Southern California
EMAIL: valouev@usc.edu
PHONE: (323) 442-7799

Thank you for your consideration

Sincerely,

Christina Boucher

Assistant Professor
University of Florida
Department of Computer and Information Science and Engineering
