# Supplementary material for: Error correcting optical mapping data
Source: Gigascience. 2018 May 25;7(6):giy061. doi: 10.1093/gigascience/giy061 (PMC6007263; doi:10.1093/gigascience/giy061)
Supplement: GIGA-D-17-00139_Revision_2.pdf [file giy061_giga-d-17-00139_revision_2.pdf]

GigaScience
Error Correcting Optical Mapping Data
--Manuscript Draft--

**Manuscript Number:**	GIGA-D-17-00139R2
**Full Title:**	Error Correcting Optical Mapping Data
**Article Type:**	Technical Note
**Funding Information:**	Division of Information and Intelligent Systems (1618814) — Dr. Christina Boucher
**Abstract:**	Optical mapping is a unique system that is capable of producing high-resolution, high-throughput genomic map data that gives information about the structure of a genome (Schwartz et al., Science 1993). Recently it has been used for scaffolding contigs and assembly validation for large-scale sequencing projects, including the maize (Zhou et al., PLoS Genetics, 2009), goat (Dong et al., Nature Biotech. 2013), and amborella (Chamala et al., Science 2013) genomes. However, a major impediment in the use of this data is the variety and quantity of errors in the raw optical mapping data, which are called Rmaps. The challenges associated with using Rmap data—and thus, optical mapping data—is analogous to dealing with insertions and deletions in the alignment of long reads. Moreover, they are arguably harder to tackle since the data is integral and susceptible to inaccuracy. We develop cOMet to error correct Rmap data, which to the best of our nowledge is the only optical mapping error correction ethod. Our experimental results demonstrate that cOMet orrects 82.49% of insertion errors and 77.38% of deletion errors in Rmap data generated from the E. coli K-12 reference genome. Out of the deletion errors corrected, 98.26% are true errors. Similarly, out of the insertion errors corrected, 82.19% are true errors. It also successfully scales to large genomes , improving the quality of 78% and 99% of the Rmaps in the plum and goat genomes, respectively. Lastly, we show the utility of error correction by demonstrating how it improves the assembly of Rmap data. Error corrected Rmap data results in an assembly that is more contiguous, and covers a larger fraction of the genome.
**Corresponding Author:**	Kingshuk Mukherjee<br>University of Florida<br>Gainesville, Florida UNITED STATES
**Corresponding Author Secondary Information:**	
**Corresponding Author's Institution:**	University of Florida
**Corresponding Author's Secondary Institution:**	
**First Author:**	Kingshuk Mukherjee
**First Author Secondary Information:**	
**Order of Authors:**	Kingshuk Mukherjee
	Darshan Washimkar
	Martin Muggli
	Leena Salmela
	Christina Boucher
**Order of Authors Secondary Information:**	
**Response to Reviewers:**	March 6, 2018<br><br>RE: GIGA-D-17-00139R1<br>Error Correcting Optical Mapping Data<br>GigaScience

Powered by Editorial Manager® and ProduXion Manager® from Aries Systems Corporation

Kingshuk Mukherjee; Darshan Washimkar; Martin Muggli; Leena Salmela; Christina Boucher

Dear GigaScience Editorial Board:

We have revised our manuscript "Error Correcting Optical Mapping Data" (GIGA-D-17-00139R1) based on the assessment of both reviewers. Please see our response to their reports below.

Sincerely,
Kingshuk Mukherjee
PhD Candidate,
Department of Computer and Information Science and Engineering,
University of Florida
Gainesville, FL

Reviewer #1:

Dear Authors,

Thank you for addressing all the comments through this revision.

I only have one query regarding the new results added to the manuscript related to the assembly of corrected, uncorrected and error-free optical maps. In Table 2, it seems that the assembly of error-free optical maps have higher N50 than the assembly of error-corrected optical maps. Why is that the case? Some text highlighting why the error-corrected data performs better assembly than error-free data will be helpful.

Response: Thank you for your comment. The N50 is akin to the mean sequence length. In this case, the N50 is calculated for three numbers (error-free Rmaps) and two numbers (corrected Rmaps), respectively, and thus, not very informative due to the small sample size. Considering the number of assembled Rmaps and their lengths, we would argue that the assembly of the error-free and corrected Rmaps is fairly equal.

Reviewer #2:

The authors have addressed many of my original comments but I believe one major one did not get the attention it deserves. This is therefore a limited review that is focussed on just this issue as I believe it affects the generality of the method and its applicability across datasets.

This is the question of the simulated datasets used here and whether or not they are realistic in light of the stated goal to develop a general method that works with optical mapping data. The authors currently use one model that was developed for data from the BioNano system (even in this model, I don't see why the parameters cannot be varied to make the data harder to analyse). However, there are other models developed for other kinds of optical mapping data which have different parameters in their model for properties like the number of false cuts or missing cuts in a typical restriction map. The authors seem to find in their analysis that the real maps they use do not have properties similar to the simulated maps they work with. Without simulating maps that resemble real maps more, it would be hard to assess what the expected performance of the method would be on such real datasets. The authors correctly note that harder datasets would elicit worse performance. However, this is not the point. What we would want to see is that there is some level of robustness in the method and that results on harder datasets are not completely useless.

Response: Thank you for your comment. We have addressed this comment in the following manner:

Powered by Editorial Manager® and ProduXion Manager® from Aries Systems Corporation

	In addition to our simulated data, we simulated data using OMSim (Miclotte et al., Bioinformatics, 2017). When we began this work there was not a third-party optical map simulator and therefore, we had to write our own. We corrected this limitation in the latest version of our manuscript.<br><br>Using OMSim, we varied the parameters by several orders of magnitude above and below the default setting for OMSim. See the "Datasets" section for a complete discussion of the parameters and our variation from the default. Tables 6 and 7 show the performance of our method on these datasets. As you can see, our method still achieves a significant improvement in the error rate of the data.<br><br>Lastly, we added results for BioNano data generated from a human sample. Again, we demonstrate the utility of our method.<br><br>We do want to note that current BioNano data is far less noisy in all regards than previous optical mapping datasets. This can be witnessed by considering the histograms of the S-scores. Hence, the Plum and Goat datasets are actually the hardest datasets from an assembly and error correction perspective.
**Additional Information:**	
**Question**	**Response**
Are you submitting this manuscript to a special series or article collection?	No
**Experimental design and statistics**<br><br>Full details of the experimental design and statistical methods used should be given in the Methods section, as detailed in our Minimum Standards Reporting Checklist. Information essential to interpreting the data presented should be made available in the figure legends.<br><br>Have you included all the information requested in your manuscript?	Yes
**Resources**<br><br>A description of all resources used, including antibodies, cell lines, animals and software tools, with enough information to allow them to be uniquely identified, should be included in the Methods section. Authors are strongly encouraged to cite Research Resource Identifiers (RRIDs) for antibodies, model organisms and tools, where possible.<br><br>Have you included the information requested as detailed in our Minimum Standards Reporting Checklist?	Yes
**Availability of data and materials**	Yes

Powered by Editorial Manager® and ProduXion Manager® from Aries Systems Corporation

All datasets and code on which the conclusions of the paper rely must be either included in your submission or deposited in publicly available repositories (where available and ethically appropriate), referencing such data using a unique identifier in the references and in the "Availability of Data and Materials" section of your manuscript.<br><br>Have you have met the above requirement as detailed in our Minimum Standards Reporting Checklist?	

Powered by Editorial Manager® and ProduXion Manager® from Aries Systems Corporation

main tex file

Click here to download Manuscript main.tex

OXFORD (GIGA)^n SCIENCE

GigaScience, 2017, 1–11

doi: xx.xxxx/xxxx
Manuscript in Preparation
Paper

PAPER

Error Correcting Optical Mapping Data

Kingshuk Mukherjee[1,*], Darshan Washimkar[2], Martin D. Muggli[2], Leena Salmela[3] and Christina Boucher[1,*]

[1]Department of Computer and Information Science and Engineering, University of Florida, Gainesville and [2]Department of Computer Science, Colorado State University, Fort Collins and [3]Department of Computer Science, Helsinki Institute for Information Technology HIIT, University of Helsinki

[*]kingdgp@ufl.edu; cboucher@cise.ufl.edu

Abstract

Optical mapping is a unique system that is capable of producing high-resolution, high-throughput genomic map data that gives information about the structure of a genome [21]. Recently it has been used for scaffolding contigs and assembly validation for large-scale sequencing projects, including the maize [32], goat [6], and amborella [4] genomes. However, a major impediment in the use of this data is the variety and quantity of errors in the raw optical mapping data, which are called Rmaps. The challenges associated with using Rmap data are analogous to dealing with insertions and deletions in the alignment of long reads. Moreover, they are arguably harder to tackle since the data is numerical and susceptible to inaccuracy. We develop cOMet to error correct Rmap data, which to the best of our knowledge is the only optical mapping error correction method. Our experimental results demonstrate that cOMet has high prevision and corrects 82.49% of insertion errors and 77.38% of deletion errors in Rmap data generated from the *E. coli* K-12 reference genome. Out of the deletion errors corrected, 98.26% are true errors. Similarly, out of the insertion errors corrected, 82.19% are true errors. It also successfully scales to large genomes, improving the quality of 78% and 99% of the Rmaps in the plum and goat genomes, respectively. Lastly, we show the utility of error correction by demonstrating how it improves the assembly of Rmap data. Error corrected Rmap data results in an assembly that is more contiguous, and covers a larger fraction of the genome.

Key words: Optical mapping; Error correction.

Introduction

In 1993 Schwartz et al. developed *optical mapping*, a system for creating an ordered, genome-wide, high-resolution restriction map of a given organism's genome. Since this initial development, genome-wide optical maps have found numerous applications including discovering structural variations and rearrangements [24], scaffolding and validating contigs for several large sequencing projects [7, 9, 4], and detecting misassembled regions in draft genomes [16]. Thus, optical mapping has assisted in the assembly of a variety of species–including various prokaryote species [18, 29, 30], rice [31], maize [32], mouse [5], goat [7], parrot [9], and *amborella trichopoda* [4]. The raw optical mapping data is generated by a biological experiment in which large DNA molecules cling to the surface of a microscope slide using electrostatic charge and are digested with one or more restriction enzymes. The restriction enzymes cut the DNA molecule at occurrences of the enzyme's recognition sequence, forming a number of DNA fragments. The fragments formed by digestion are painted with a fluorescent dye, to allow visibility under laser light and a CCD camera. Computer vision algorithms then estimate fragment length from consolidated intensity of fluorescent dye and apparent distance between fragment ends.

The resulting data from an experiment are in the form of an ordered series of fragment lengths [33]. The data for each single molecule produced by the system is called an *Rmap*. Rmap data has a number of errors due to the experimental conditions and system limitations. In an optical mapping experiment, it is unlikely to achieve perfectly uniform fluorescent staining.

Compiled on: March 6, 2018.
Draft manuscript prepared by the author.

This leads to an erroneous estimation of fragment sizes. Also, restriction enzymes often fail to digest all occurrences of their recognition sequence across the DNA molecule. This manifests as missing restriction sites. Additionally, due to the fragile nature of DNA, additional breaks can incorrectly appear as restriction sites. Lastly, the limitations of the imaging component of the optical mapping system and the propensity for the DNA to ball up at the ends introduces more sizing error for smaller fragments. Interested readers will find more details about the causes of these errors in Valouev *et al.* [26] and Li *et al.* [12]. Because of all these experimental conditions, Rmap data generated through optical mapping experiment has insertion (added cut sites) and deletion (missed cut sites) errors along with fragment sizing errors.

In most applications of optical map data, the Rmaps need to be assembled into a genome wide optical map. This is because the single molecule maps need redundant sampling to overcome the presence of the aforementioned errors, and because single molecule maps only span on the order of 500 Kbp [26]. The first step of this assembly process involves finding pairwise alignments amongst the Rmaps. In order to accomplish this, the challenge of dealing with missing fragment sizes has to be overcome. This challenge is analogous to dealing with insertions and deletions in the alignment of long reads [2]—in fact, it is arguably harder since the data is numerical. At present, the only non-proprietary algorithmic method for pairwise alignment of Rmaps is the dynamic programming based method of Valouev *et al.* [26] which runs in $O(\alpha \times \beta)$ time where α and β are the number of fragments in the two Rmaps being aligned. To align an optical map dataset containing n Rmaps, the complexity becomes $O(n^2 \times \ell^2)$ where ℓ is the average size of an Rmap.

This method is inherently computationally intensive but if the error rate of the data could be improved then non-dynamic-programming based methods that are orders of magnitude faster such as Twin [15], OMBlast [10], and Maligner [13] could be used for alignment. This would greatly improve the time required to assemble Rmap data. Thus, we present cOMet in order to address this need. To the best of our knowledge, it is the first Rmap error correction method. Our experimental results demonstrate that cOMet has high precision and corrects 82.49% of insertion errors and 77.38% of deletion errors in Rmap data generated from the *E.coli* K-12 reference genome. Out of the deletion errors corrected, 98.26% are true errors. Similarly, out of the insertion errors corrected, 82.19% are true errors. Furthermore we show that the assembly of Rmaps is more contiguous and covers a larger fraction of the genome if the Rmaps are first error corrected. It also successfully scales to large genomes, improving the quality of 78% and 99% of the Rmaps in the plum and goat genome, respectively.

Background

From a computer science perspective, optical mapping can be seen as a process that takes in two strings: a nucleotide sequence $S_i[1, n]$ and a restriction sequence $B[1, b]$, and produces an array (string) of integers $R_i[1, m]$. The array R_i is an Rmap corresponding to S_i and contains the string-lengths between cuts produced by B on S_i. Formally, R_i is defined as follows: $R_i[j] = y - x$ where y represents the location(starting index) of jth occurrence of B in S_i and x represents the location of $(j-1)^{th}$ occurrence of B in S_i and $R_i[1] = y - 1$ and $R_i[m] = n - x$. For example, say we have $B = act$ and $S_i = atacttactggactactaaact$. The locations of B in S_i are as follows: 3,7,12,15,20. Then R_i will be represented as $R_i = 2, 4, 5, 3, 5, 2$. The size of an Rmap denotes the number of fragments in that Rmap. Therefore the size of R_i is 6.

We note that millions of Rmaps are produced for a single genome since optical mapping is performed on many cells of the organism and each cell provides thousands of Rmaps. The Rmaps can be assembled to produce a genome wide optical map. This is analogous to next generation shotgun sequencing where Rmaps are analogous to reads and a genome-wide optical map is analogous to the assembled whole genome.

There are three types of errors that can occur in optical mapping: (1) missing cut sites which are caused by an enzyme not cleaving at a specific site, (2) additional cut sites which can occur due to random DNA breakage and (3) inaccuracy in the fragment size due to the inability of the system to accurately estimate the fragment size. Continuing again with the example above, a more representative example Rmap would include these errors, such as $R_i' = 7, 6, 3, 4$.

The error rates of optical maps depends on the platform used for generating the maps. A recent paper by Li *et al.* [12] studied the error rates of optical maps produced by the Irys system from BioNano Genomics. According to their study, a missing cut site type of error i.e., error type (1) happens when a restriction site is incompletely digested by the enzyme and causes two flanking fragments to merge into one large fragment. The probability of complete digestion of a restriction site can be modeled as a Bernoulli trial whose probability of success is a function of the size of the two flanking fragments. Additional cut sites i.e., error type (2) results from random breaks of the DNA molecule. The number of false cuts per unit length of DNA follows a Poisson distribution. The inaccuracy of the fragment sizes, i.e., error type (3), is modeled using a Laplace distribution. If the observed and actual size of a fragment are o_k and r_k respectively, then the sizing error is defined as $s_k = o_k/r_k$ and

$$s_k \sim Laplace(\mu, \beta)$$

where μ and β – the parameters of the laplace distribution – are functions of r_k. In practice, when aligning a pair of Rmaps, one should allow for twice the error rate of a single Rmap since each Rmap will deviate from the genomic map by the above parameters.

Valouev *et al.* [26] provides a dynamic programming algorithm for pairwise alignment, which generates a score for every possible alignment between two Rmaps and returns the alignment which achieves the highest score, which is referred to as the S-score. It is computed within a standard dynamic programming framework, similar to Smith-Waterman alignment [23]. The scoring function is based on a probabilistic model built on the following assumptions: the fragment sizes follow an exponential distribution, the restriction sites follow an independent Bernoulli process, the number of false cuts in a given genomic length is a Poisson process, and the sizing error follows a normal distribution with mean zero and variance following a linear function of the true size. Lastly, a different sizing error function is used for fragments less than 4 kbp in length since they do not converge to the defined normal distribution. The score of an alignment is calculated as the sum of two functions; one function that estimates and scores the sizing error, and a second that predicts and scores the presence of additional and/or missing cut sites between the fragments. The S-score will be used later in this paper to evaluate the error correction process.

Methods

Given a set of n Rmaps $\mathsf{R} = \{R_1, .., R_n\}$ our method aims to detect and correct all errors in R by considering each $R_i \in \mathsf{R}$ and finding a set of Rmaps that originate from the same part of the

genome as R_i. This step is performed heuristically in order to avoid aligning every pair of Rmaps in R.

Preprocessing

Our first step is to remove the first and last fragments from each Rmap in R. These fragments have one of their edges sheared by artifacts of the DNA prep process (preceding the optical mapping process) and not by restriction enzymes. Unless removed, they can misguide alignment between two Rmaps during the error correction process. In addition, short Rmaps, i.e., those that have less than 10 fragments, are removed at this stage since any Rmap that contains less than 10 fragments is typically deemed too small for analysis even in consensus maps [1]. Next, the data is quantized so that a given genomic fragment is represented by the same value across multiple Rmaps despite the noise. Our quantization method assigns a unique value to a range of fragment sizes by dividing each fragment size by a fixed integer, denoted as b, and rounding to the nearest integer. For example, if an Rmap $R_i = \{36, 13, 15, 20, 16, 5, 21, 17\}$ is quantized using $b = 3$ then the quantized Rmap will be $R_i^{quantized} = \{12, 4, 5, 7, 5, 2, 7, 6\}$. Say another Rmap, $R_j = \{17, 23, 34, 12, 14, 21, 14, 5\}$ has overlap with R_i; however, due to noise in the data, this relation is not apparent. By quantizing R_j using the same $b = 3$ we get $R_j^{quantized} = \{6, 8, 11, 4, 5, 7, 5, 1\}$. This allows us to uncover a region (in this case $\{4, 5, 7, 5\}$) which is common to both the Rmaps. It should be noted that in some cases, a fragment may have different values across two Rmaps even after quantization (for example the fragment values 36 from R_i and 34 from R_j are quantized to 12 and 11 respectively). The quantized data is used to find the set of *related* Rmaps as explained in the next section.

The setting of parameter b depends on the amount of sizing error in the optical map data. With zero sizing error b can be set at 1. As sizing error increases, the value of b is increased accordingly. If the value of b is too small, we are not be able to uncover relations between overlapping Rmaps and if the value is too large then unrelated Rmaps have common regions in their quantized states — which makes them appear related. Considering the error rate of optical maps from BioNano genomics, the default value of $b = 4000$.

Finding Related Rmaps

We refer to two Rmaps as *related* if their corresponding error-free Rmaps originate from overlapping regions of the genome. Next, we define a k-mer as a string of k consecutive fragments from a (quantized) Rmap. For example if we have the Rmap $R = \{3, 3, 5, 2, 6, 5, 5, 1\}$ and k=4 then the following k-mers can be extracted from R: (3,3,5,2), (3,5,2,6), (5,2,6,5), (2,6,5,5) and (6,5,5,1). In order to avoid aligning all pairs of Rmaps to find the related Rmaps, we use the number of common k-mers to discriminate between pairs of Rmaps that are related and those that are not. To accomplish this efficiently, we first extract all unique k-mers in each quantized Rmap, and construct a hash table storing each unique k-mer as a key and the list of Rmaps containing an occurrence of that k-mer as the value. We call this the k-mer index. Next, we consider each R_i in R and use the k-mer index to identify the set of Rmaps that have m or more k-mers in common with R_i. Unfortunately, this set, although it contains all related Rmaps, it also likely contains Rmaps that are not related to R_i. Therefore, we filter this set of Rmaps using a simple heuristic that tries to match each Rmap in this set with R_i in order to ascertain if it is related to R_i. The heuristic traverses through two Rmaps (R_i and one Rmap from the set, say R_j) attempting to match subsets of the fragments from each until it either reaches the end of one Rmap or it fails to match the fragments. We start the traversal from the first matching k-mer between R_i and R_j. We denote the position of the next fragment to be matched in R_i and R_j as x and y, respectively, and assume that each fragment prior to these positions is matched. Next, we consider all combinations of matching the fragments at positions x, $x+1$ and $x+2$ of R_i with fragments at positions y, $y+1$ and $y+2$ of R_j. We evaluate the cost of each combination based on the difference in the total size of fragments from R_i and R_j. That is $\forall \alpha, \beta = [0, 2]$,

$$cost(x + \alpha, y + \beta) = \left| \sum_{g=x}^{x+\alpha} R_i[g] - \sum_{h=y}^{y+\beta} R_j[h] \right|$$

where $R_i[g]$ and $R_j[h]$ denotes the g-th and h-th fragments of R_i and R_j respectively. We select the combination with the least cost; if there exists a tie, we select the match that has the least number of added or missing cut sites (That is, the combination with the least value of $\alpha + \beta$). If this selected match leads to a cost that is greater than a specified threshold (which was set to 25% of the larger sized fragment in practice), then we conclude that there is not a match at these positions and return that R_i and R_j are unrelated. Otherwise, we increment x and y accordingly and move onto the next fragments. If this heuristic continues until the last fragment of either R_i or R_j is reached then we return that R_i and R_j are related. Using this heuristic we filter out the Rmaps that were deemed to be related based on the number of k-mers in common with R_i but are infact unrelated to R_i.

The setting of parameters k and m are correlated. If the value of k is increased, that makes the k-mers more specific, hence, the value of m is lowered. On the other hand, if the value of k is reduced, then we increase the value of m. The value of k is increased when there are fewer insertion and deletion errors and decreased otherwise. The default values are $k = 4$ and $m = 1$.

Rmap Alignment

Next, for each R_i in R, we use the alignment method of Valouev *et al.* [26] to find the S-score of all pairwise alignments between R_i and each Rmap in its set of related rmaps. The Rmaps that have an alignment score, i.e., S-score less than a defined threshold (which we denote as S_t), are removed from the set of related Rmaps and the alignments of the remaining Rmaps are stored in a *multiple alignment grid*, denoted as A_i. This grid is a two-dimensional array of integer pairs, where the number of rows is equal to the number of remaining Rmaps in the set of related Rmaps of R_i and the number of columns is equal to the number of fragments in R_i. An element of this array, $A_i[j, k]$ stores an integer pair in the form of (x, y) representing that x fragments of R_i, (which includes the k-th fragment of R_i) matches to y fragments of R_j in the optimal alignment between R_i and R_j. Figure 1 illustrates an example of A_i. The first fragment of R_i does not match with any fragment of R_j and therefore, (0, 0) is stored at this position. Fragments 2, 5, 6, 8 and 9 of R_i each matches with one fragment of R_j, e.g., 1, 3, 4, 7 and 8, respectively. To represent these matches, we store a (1,1) in 2nd, 5th, 6th, 8th and 9th column of row j. Fragments 3 and 4 of R_i match with one fragment of R_j, i.e., the 2nd fragment. To represent this, we store (2,1) in $A_i[j, 3]$ and $A_i[j, 4]$. Fragment 7 of R_i matches with two fragments of R_j, i.e., the 5th and 6th fragments. To represent this, we store (1,2) in $A_i[j, 7]$. Fragments 10 and 11 of R_i match with two fragments of R_j, i.e., the 9th and 10th fragments. To represent this, we store (2,2) in positions $A_i[j, 10]$ and $A_i[j, 11]$. Finally, fragments 12 and 13 match with three fragments of R_j, i.e., fragments 11, 12 and 13. In this case, we store (2,3) in positions $A_i[j, 12]$ and $A_i[j, 13]$.

	1st	2nd	3rd	4th	5th	6th	7th		8th	9th	10th	11th	12th	13th	
R_i	1.474	3.625	2.092	2.164	8.424	2.331	24.824		7.267	2.954	12.578	2.358	8.955	22.943	
R_j		3.331	4.464		8.287	2.481	10.314	13.391	7.711	3.143	8.448	5.921	13.795	4.143	6.119

A_i	1st	2nd	3rd	4th	5th	6th	7th	8th	9th	10th	11th	12th	13th
:	:	:	:	:	:	:	:	:	:	:	:	:	:
j	(0,0)	(1,1)	(2,1)	(2,1)	(1,1)	(1,1)	(1,2)	(1,1)	(1,1)	(2,2)	(2,2)	(2,3)	(2,3)
:	:	:	:	:	:	:	:	:	:	:	:	:	:

Figure 1. An alignment between R_i and R_j as given by Valouev *et al.* [26] and its corresponding entry in the multiple alignment grid A_i. Each column of A_i represents one fragment from R_i and each row represents one Rmap from its' set of related Rmaps. The fragment sizes are in Kbp.

The setting of parameter S_t controls the number of Rmaps that are included in the multiple-alignment-grid of an Rmap. If we increase the value of S_t, fewer Rmaps will be added to the grid — but the ones included will be of higher quality (i.e. have greater overlap with the Rmap under consideration). The default value for the parameter $S_t = 8$. We show in the experiment section how we select this value.

Error Correcting Using the Consensus

The multiple alignment grid is used to find the consensus grid, denoted as C_i, for Rmap R_i. The grid C_i is a one-dimensional array of integer pairs with size equal to the number of fragments in R_i. The grid is constructed for each R_i in R by iterating through each column of A_i and finding the most frequent integer pair, breaking ties arbitrarily. The most frequent integer-pair is stored at each position of C_i if the frequency is above a given threshold d; otherwise, (0,0) is stored. Figure 2 illustrates the construction of a consensus grid from an alignment grid. The type of error in each fragment of R_i can be identified using $C_i[k] = (x, y)$ as follows: if x and y are equal then a sizing error occurs at the k-th fragment of R_i, otherwise, if x is greater than y then an additional cut site exists, and lastly, if x is less than y then a missing cut site exists. Next, we use C_i and A_i to correct these errors in R_i. For each fragment of R_i, we consider the consensus stored at the corresponding position of C_i, identify the positions in the corresponding column of A_i that are equal to it, and replace the fragment of R_i with the mean total fragment size computed using the values at those positions in A_i. If C_i is equal to (0,0) at any position then the fragment at that position in R_i remains unchanged since it implies that there is no definitive result about the type of error in that position. In addition, if consecutive positions in C_i are discordant then the fragments in those positions in R_i also remains unchanged. For example, if there is a (2,1) consensus at some position of C_i, then we expect the preceding or successive position to also have a (2,1) consensus. However, if this is not the case, then we do not error correct those fragments since the consensus is discordant at those positions. Figure 2 shows this error correction. As it is illustrated, to error correct the second fragment of R_i, we compute the average of the matched fragments from related Rmaps 2, 3, 4, 5 and 6 and replace the second fragment of R_i with that value as shown in Figure 2. Similarly, to correct the third fragment in this example, we identify that (2,1) is in the consensus, which implies that majority of the related Rmaps are such that two fragments of R_i match with one fragment from the set of related Rmaps, and therefore, replace the third and fourth fragments with the average from the corresponding Rmaps and positions.

The threshold d determines the accuracy and precision of error correction. A high value of d improves precision but lowers accuracy as many fragments are left uncorrected. Similarly, low value of d improves accuracy but lowers precision. The default setting is $d = 3$.

Complexity

We define ℓ to be the length of the longest Rmap in R. Quantization of the Rmaps takes $O(\ell \times n)$ time. Constructing the k-mer index also takes $O(\ell \times n)$ time. The k-mer index stores the occurances of each quantized k-mer across all Rmaps. Let u be maximum frequency of a k-mer. That is, a k-mer occurs in max u Rmaps (in practice $u << n$). Then the complexity of finding related Rmaps from the k-mer index is $O(n \times \ell \times u)$. For each Rmap, the filtering heuristic runs in time linear to the size of the Rmap. Therefore, filtering the set of related Rmaps also takes linear $O(\ell \times n)$ time. The most expensive step is the pair-wise alignment which uses the Valouev aligner. As mentioned earlier, this aligner is based on DP and therefore has a $O(\ell^2)$ time complexity to perform one pairwise alignment. If the maximum cardinality of the set of related Rmaps for any Rmap is v, then the total complexity of this step is bounded by $O(n \times v \times \ell^2)$. The value of v depends on the coverage of the optical map data. The alignment generated using Valouev *et al.* method is stored in the multiple alignment grid in constant time and it takes $O(n \times v \times \ell)$ time to generate the consensus maps for n Rmaps and error correct them. Thus, the runtime of cOMet is $O(n \times v \times \ell^2)$.

Datasets

We perform experiments on both simulated and real data. For the real data, we used the Rmap data from the plum [28] and domestic goat [7] sequencing projects. These datasets were built on the OpGen mapping platform and are more error-prone. We also experimented on a human dataset [22] built on the new BioNano platform. This dataset is built using the latest optical mapping technology and has significantly better quality than the plum and goat genomes.The genome size and number of Rmaps for these species are shown in Tables 1 and 1.

In addition, we simulated Rmap data from *E. coli* K-12 substr. MG1655 as follows: first, the reference genome was copied 200 times and then uniformly distributed random loci were selected for each of these copies. These loci form the ends of

Multiple alignment grid (A_i)

A_i	1st	2nd	3rd	4th	5th	6th	7th	8th	9th	10th	11th	12th	13th
1	(1,1)	(2,2)	$(2,2)_{\{2.168,3.511\}}$	$(1,1)_{\{2.107\}}$	$(2,1)_{\{10.221\}}$	(2,1)	(1,2)	(1,1)	(1,1)	(2,2)	(2,2)	(1,1)	(1,2)
2	(0,0)	(1,1)	$(2,1)_{\{4.344\}}$	$(2,1)_{\{4.344\}}$	$(1,1)_{\{8.488\}}$	(1,1)	(1,2)	(1,1)	(1,1)	(2,2)	(2,2)	(2,3)	(2,3)
3	(0,0)	(1,1)	$(2,1)_{\{4.129\}}$	$(2,1)_{\{4.129\}}$	$(1,1)_{\{8.132\}}$	(1,1)	(1,2)	(1,1)	(1,1)	(1,1)	(1,1)	(1,1)	(1,2)
4	(0,0)	(1,1)	$(2,1)_{\{4.311\}}$	$(2,1)_{\{4.311\}}$	$(1,1)_{\{8.964\}}$	(1,1)	(1,2)	(1,1)	(1,1)	(2,2)	(2,2)	(2,3)	(2,3)
5	(1,1)	(1,1)	$(2,1)_{\{4.611\}}$	$(2,1)_{\{4.611\}}$	$(2,1)_{\{10.692\}}$	(2,1)	(3,3)	(3,3)	(3,3)	(1,1)	(1,1)	(1,1)	(1,2)
6	(0,0)	(1,1)	$(2,1)_{\{4.710\}}$	$(2,1)_{\{4.710\}}$	$(1,1)_{\{9.432\}}$	(1,1)	(1,2)	(1,1)	(1,1)	(2,2)	(2,2)	(2,3)	(2,3)

Consensus grid (C_i)

C_i	(0,0)	(1,1)	(2,1)	(2,1)	(1,1)	(1,1)	(1,2)	(1,1)	(1,1)	(2,2)	(2,2)	(1,1)	(1,2)

Error correction

R_i	1.474	3.625	2.092	2.164	8.424	2.331	24.824		7.267	2.954	12.578	2.358	8.955	22.943	
C_i	(0,0)	(1,1)	(2,1)	(2,1)	(1,1)	(1,1)	(1,2)		(1,1)	(1,1)	(2,2)	(2,2)	(1,1)	(1,2)	
R'_i	1.474	3.273	4.421		8.754	2.608	9.988	13.891	7.184	3.472	8.532	6.032	10.038	5.633	15.869

Figure 2. Example of multiple alignment grid and consensus grid. The figure shows the multiple alignment grid A_i for an Rmap R_i and its consensus grid C_i. Each row of the multiple alignment grid represents the alignment of R_i with one of its related Rmaps while the columns represents the fragments of R_i. The figure also demonstrates error correction using the consensus grid, with the error corrected Rmap denoted as R'_i. The fragment sizes are in Kbp. To demonstrate the error correction process for the 3rd, 4th and 5th fragments, we also include the fragments (in parentheses) to which they align. The error corrected fragment is the mean of the fragments from the corresponding positions which have the same alignment as the consensus. For example for the 5th fragment, the consensus is (1,1). Therefore the mean of the aligned fragments with (1,1) alignment i.e. 8.488, 8.132, 8.964 and 9.432 is the error-corrected value for the 5th fragment.

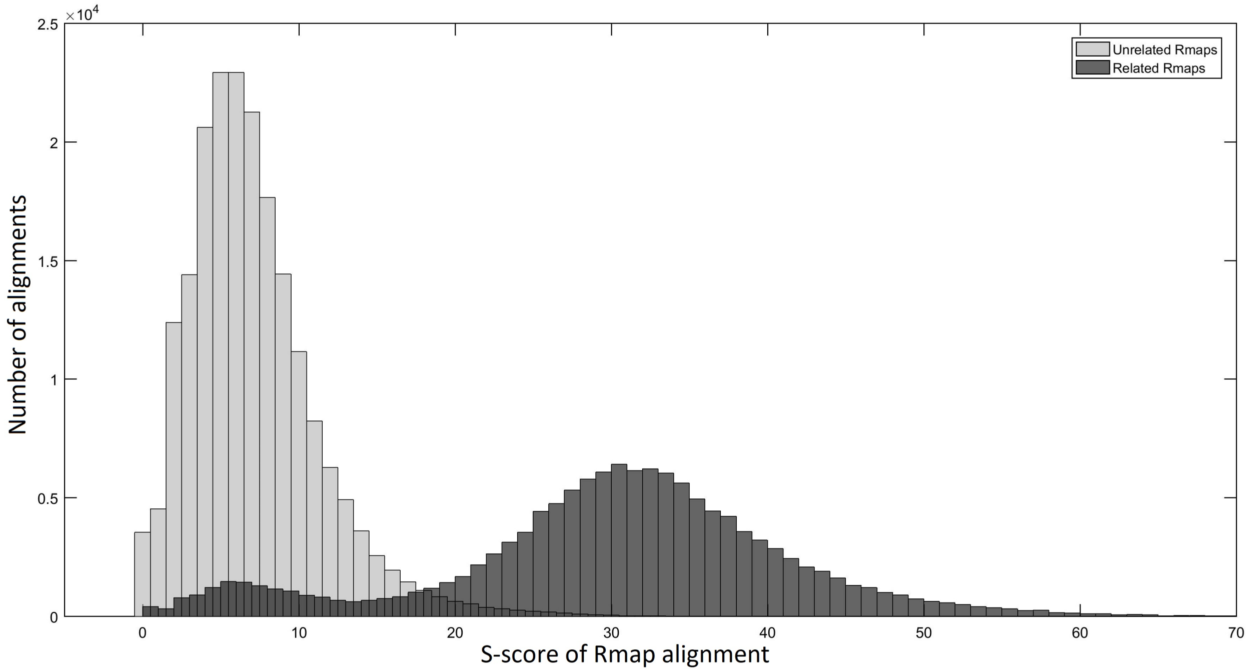

Figure 3. Distribution of S-scores of Rmap alignments between related Rmaps and unrelated Rmaps. The percentage of Related Rmaps with S-score less than 8 is 6.06%. Therefore we choose S_t = 8.

single molecule that would undergo *in silico* digestion. Next, molecules smaller than 150 Kbp were discarded and the cleavage sites for the RsrII enzyme were then identified within each of these simulated molecules. This error free Rmap data is used for validating the output of our method. Lastly, deletion, insertion and sizing errors were incorporated into the error-free Rmaps according to the error model discussed in Li *et al.* [12]. The error model was described earlier in the Background section. This simulation resulted in 2,505 Rmaps, containing 7,485 deletion and 554 insertion errors.

Lastly, we simulated optical map data from a simulation software called OMSim [14] that generates synthetic optical maps which mimics real Bionano Genomics data. The software takes two parameters as input: the False Positive Rate (FP), which is the number of additional cut sites erroneously inserted per 100kbp, and the False Negative Rate (FN), which is the percentage of times a cut site is missed. Using this method, we simulated eight datasets of Rmaps from *E. coli* K-12 substr. MG 1655 using the restriction enzyme BspQI. The default FP and FN rates for BspQI are 1 and 15% respectively.

We generated additional datasets with the following error rates (FP,FN) : (0.5,15%), (1.0,15%), (2.0,15%),(5.0,15%), (1.0,5%), (1.0,25%),(2.0,5%) and (2.0,25%).

Table 1. Summary of the real and simulated data. Rmaps with less than 10 fragments were omitted from all the experiments. cOMet was ran on the remaining 2,504, 548,779 and 3,049,439 Rmaps for the E. coli, plum and goat genomes, respectively..

Genome	Size	No. of Rmaps
E. coli	4.6 Mbp	2,504
Plum	284 Mbp	749,895
Goat	2.66 Gbp	3,447,997

Table 2. Summary of the real and simulated BioNano data. OMSim was used to simulate eight different BioNano datasets; each of which had varying error rates and thus, had a different number of Rmaps.

Genome	Size	No. of Rmaps
E. coli	4.6 Mbp	123,251 - 157,743
Human	3.2 Gbp	793,199

Experiments and Discussion

We performed all experiments on Intel E5-2698v3 processors with 192 GB of RAM running 64-bit Linux. The input parameters to cOMet include: b (quantization bucket size), k (k-mer value), m (the number of k-mers needed to be conserved between two Rmaps) and d (the minimum number of Rmaps required to form consensus at a position). The default parameters are b=4000, k=4, m=1 and d=3, and led to the best result across all datasets.

Determining the value of S_t

The setting of the parameter S_t depends on the sensitivity of the Valouev aligner. If the alignment score between two Rmaps is less than S_t, then the aligned Rmaps are deemed to be unrelated. We say an Rmap, R_s is *overlapping* with an Rmap, R_t if at least 50% of R_s overlaps with R_t. That is, either the first half or the second half of R_s is entirely and exactly (exact fragment matches) contained in R_t.

We carried out the following experiment to determine the optimum setting for S_t. From the set of simulated error-free Rmaps, we computed the set of overlapping Rmaps for each Rmap. We denote this set as *related Rmaps*. Then we used the Valouev aligner to score all pairwise wise alignments between the simulated Rmaps (with errors added) and plot the scores in form of a histogram which is shown in Figure 3. The percentage of related Rmaps with S-score less than 8 is 6.06%. Hence we choose the setting of S_t = 8.

Experiments with our Simulated Data

The cOMet error correction was ran on the simulated *E. coli* data. The corrected Rmaps were then aligned to the error-free Rmaps to determine the number of corrected insertions and deletions. The results of this experiment are shown in Table 4. To determine the quality of error correction, we computed the true positive rate (TPR), which is the ratio between the number of insertion (or deletion) errors that cOMet correctly identified and removed and the number of insertion (deletion) errors, and the false positive rate (FPR), which is the ratio between the number of insertion (or deletion) errors that cOMet incorrectly identified and removed, and the total number of fragments not containing an insertion (deletion) error. The TPR is 82.49% and 77.38% with respect to the number of corrected insertions and deletion errors; whereas, the FPR is 0.21% and 0.25% with respect to the number of corrected insertions and deletion errors. This demonstrates the high accuracy of the correction made by cOMet. Our method also has high precision. Out of the deletion errors corrected, 98.26% are true errors. Similarly, out of the insertion errors corrected, 82.19% are true errors.

Additionally, for each corrected Rmap we computed the alignment S-score of both the original Rmap and the corrected Rmap with the error-free Rmap. We found that for 96.5% of the Rmaps, the S-scores improved after error correction. In other words, cOMet brought 96.5% Rmaps closer to their error-free state. The mean S-score before error-correction was 44.91 and it improved by 14.03% to 51.30 after error correction. For 17.5% of the Rmaps, (415 Rmaps) the S-score improved by more than ten. Lastly, we mention that the error correction was achieved in 241 CPU seconds and using 79.54 MB of memory.

To demonstrate the importance of error correction, we assembled the Rmaps before and after error correction using the Valouev assembler [25]. Table 3 summarizes the results of this experiment. We assembled the uncorrected data into five assembled optical maps and the error-corrected data into two assembled optical maps. The N50 statistic of the assembly increased from 1,242 Kbp for the uncorrected data to 3,348 Kbp for the corrected data. Next, we aligned each assembled map to the genome-wide (error-free) optical map using the Valouev aligner in order to locate their positions on the genome and calculate the percentage of the genome that was covered by at least one of the assembled maps. The genome fraction covered by the five assembled maps from the uncorrected Rmaps was 80%; while the genome fraction covered by the two assembled maps from the corrected Rmaps was 82%. Moreover, the assembled maps from the uncorrected data had 47 insertion and deletion errors when aligned to the reference while the error corrected data had only 34 such errors. In order to further contextualize these results, we assemble the error-free Rmap dataset and summarize this assembly in Table 3.

Experiments with OMSim Data

To present the robustness of our method and its applicability across datasets, we conducted experiments on synthetic data from an optical map simulating software called OMSim [14]. As described in Section Datasets, we generated eight datasets of synthetic optical maps by varying the insertion and deletion error rates.

In the first experiment, we fixed the False Negative rate at 15% and varied the False Positive rate between 0.5, 1.0, 2.0 and 5.0 respectively. For each of the four datasets, we align each Rmap (using the Valouev aligner) before and after error correction to the reference optical map obtained using the same restriction enzyme and report the percent of Rmaps whose alignment S-score increased after error correction and the mean increase in the S-score. We note that for each Rmap, the aligner returns the highest scored alignment and the score represents how closely the Rmap aligns to the reference genome-wide optical map. Table 6 summarizes the results from this experiment. We observe that the efficiency of error-correction improves as the FP rate is initially increased. When the FP rate reaches a high value of 5, the efficiency of error-correction

Table 3. Assembly results of Uncorrected Rmaps, Corrected Rmaps and Error-free Rmaps using the Valouev assembler. The Rmaps are simulated from the *E. coli* genome. Each assembled map is aligned to the reference genome-wide (error-free) optical map using the Valouev aligner. The genome-wide optical map contains 383 fragments.

Rmap status	Assembler Map_id	Number of fragments	Map length (in Kbp)	Alignment location in reference (start-loci, end-loci)
Uncorrected Rmaps	Assembled Map_0	75	921.41	(246,321)
	Assembled Map_1	88	1,242.40	(11,95)
	Assembled Map_2	30	531.65	(225,255)
	Assembled Map_3	44	759.16	(181,228)
	Assembled Map_4	107	1,699.60	(87,194)
Corrected Rmaps	Assembled Map_0	102	1,397.60	(225,322)
	Assembled Map_1	237	3,348	(8,230)
Error-free Rmaps	Assembled Map_0	60	808.74	(185,239)
	Assembled Map_1	91	1,100.5	(241,324)
	Assembled Map_2	104	2,474.4	(19,185)

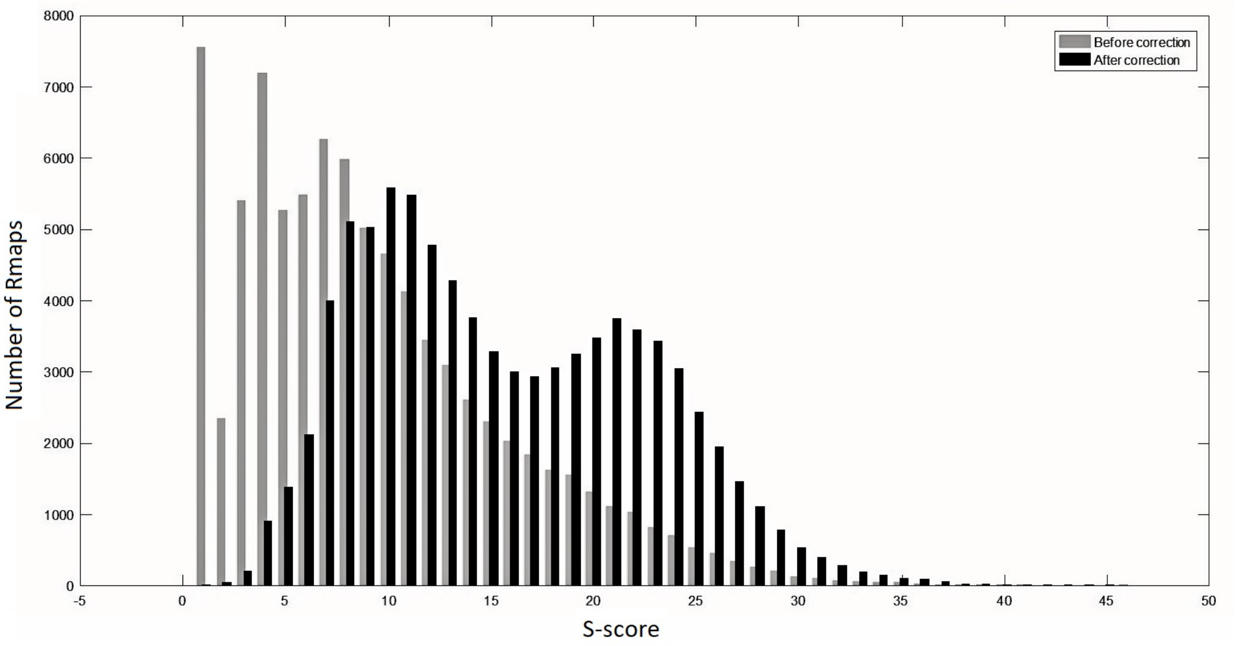

Figure 4. Alignment scores of Rmaps from plum genome with the reference optical-map. Before error correction, the *S*-score had a mean of 8.6 with standard deviation 6.49. After error correction, the mean *S*-score improved to 14.72, with standard deviation 6.72.

Table 4. Results on the data simulated from *E.coli* K-12 MG 1655. The data was simulated according the algorithm described in Datasets. This simulation resulted in 2,505 Rmaps, containing 7,485 deletion and 554 insertion errors.

Total no. of insertion errors corrected	556
TPR of corrected insertions	82.49 % (457)
FPR of corrected insertions	0.21 % (99)
Total no. of deletion errors corrected	5,894
TPR of corrected deletions	77.38 % (5,792)
FPR of corrected deletions	0.25 % (102)

Table 5. Results on the Rmap data of plum and goat genomes. Peak memory was measured as the maximum resident set size as reported by the operating system with sufficient RAM to avoid paging. Running time is the user process time, also reported by the operating system.

Genome name	Plum	Goat
Running time	7.4 days	105.7 days
Memory	12.20 GB	113.56 GB
No. of insertion errors corrected	433,282	2,530,060
No. of deletion errors corrected	430,329	3,187,023

drops. The mean S-score improves by more than 9 (~ 10%) when the FP rate is reasonable.

In the second experiment, we first fix the False Positive rate at 1.0 and vary the False Negative rate between 5%, 15% and 25%. We then fix the False Positive rate at 2 and vary the false negative rate between 5%, 15% and 25%. We report the same results as the previous experiment. Table 7 shows the results. Similar to the previous experiment, we find that the efficiency of error correction improves as the FN rate increases from 5% to 25%. The error correction improves the quality of a high percentage of Rmaps (> 70%) for all values of parameters.

Experiments with Real Data

Table 5 summaries the results of running cOMet on the plum and goat datasets. The plum and goat datasets do not contain error-free Rmaps. Therefore, we are restricted to reporting the number of corrections made and the improvement to the *S*-score. In order to compute the *S*-score before and after error correction, we generated an *in silico* digested genome-wide optical map from the reference genome and aligned both the uncorrected and corrected Rmap to the genome-wide optical map. If it aligned to multiple positions then we considered

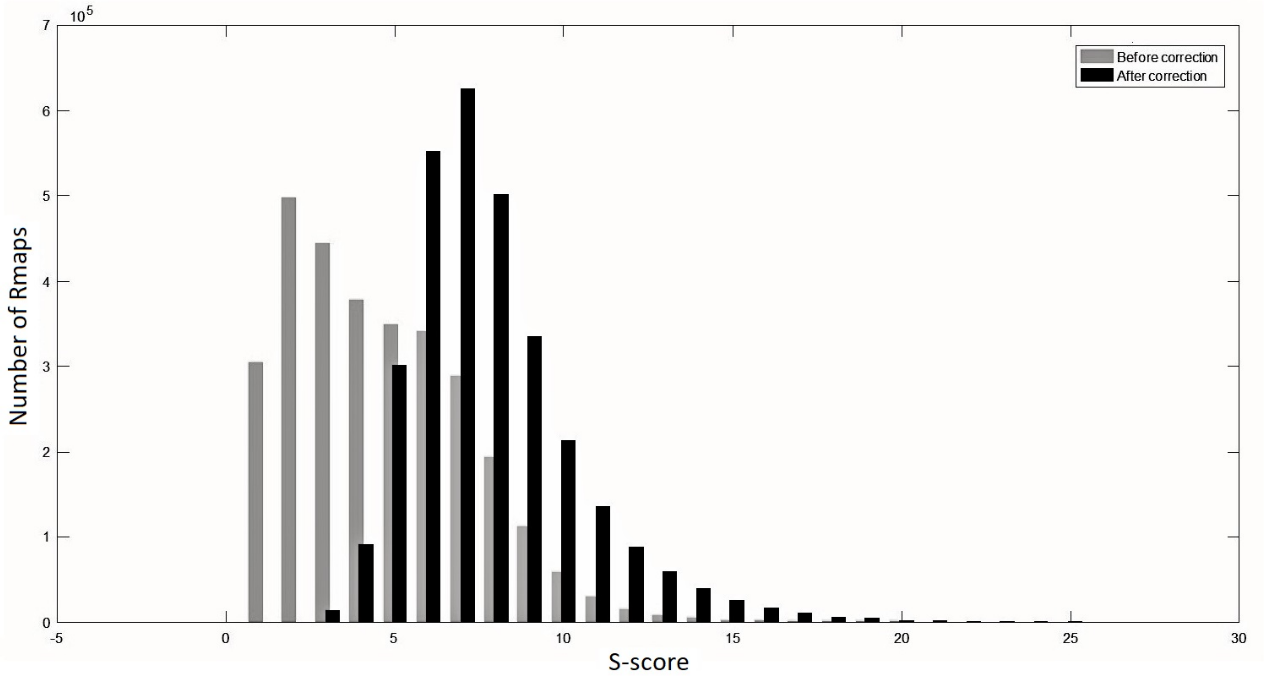

Figure 5. Alignment scores of rmaps from goat genome with the reference optical-map. The mean and standard deviation of the *S*-scores before error correction were 9.38 and 6.54, respectively. After error correction, the mean *S*-score improved to 16.97 with a standard deviation of 6.21.

Table 6. Table showing the efficiency of error correction when the False Positive rate is varied. The False Negative rate is fixed at 15%.

FP rate	No. of Rmaps	Percent of Rmaps with improved S-score	Mean S-score before error-correction	Mean S-score after error-correction	Mean S-score improvement
0.5	129,820	93.42%	78.66	91.12	12.46
1.0	126,133	94.01%	76.25	89.44	13.19
2.0	140,623	92.99%	71.93	85.29	13.36
5.0	130,019	81.35%	64.65	71.36	6.71

Table 7. Table showing the efficiency of error correction when the False Negative rate is varied.

FP rate	FN rate	No. of Rmaps	Percent of Rmaps with improved S-score	Mean S-score before error-correction	Mean S-score after error-correction	Mean S-score improvement
1.0	5%	142,684	87.36%	81.32	87.62	6.3
	15%	126,133	94.01%	76.25	89.44	13.19
	25%	123,252	96.09%	70.24	87.44	17.20
2.0	5%	148,912	89.23%	76.54	84.47	7.93
	15%	140,623	92.99%	71.93	85.29	13.36
	25%	130,763	93.02%	66.95	81.65	14.7

the alignment position where the corrected Rmap aligned with greatest *S*-score, and considered the difference in the *S*-score when the uncorrected and corrected Rmap aligned to that position. However, we note that this process is error prone because of the fragmented nature of the draft genomes and possible misassemblies present in the genomes. We observed that the *S*-score after error correction improved for 78% of the plum Rmaps and 99% of the goat Rmaps. Figures 4 and 5 show the histograms of the distribution of *S*-scores, before and after error correction. For the plum genome, the mean *S*-score improved from 8.60 before error correction, to 14.72 after error correction (a 71% improvement in the score) while for the goat genome, it improved from 9.38 before correction to 16.97 after correction (a 80.92% improvement in the score).

We also measured the *genome coverage*, i.e. the fraction of the genome covered by at least one Rmap, for both the original Rmaps and the corrected Rmaps as follows. First we aligned all Rmaps to the genome-wide optical map and then picked the best alignment for each original Rmap and each corrected Rmap. Based on these alignments we then computed the fraction of the genome covered by at least one original Rmap and the fraction of the genome covered by at least one corrected Rmap. On the goat genome the genome coverage was 73.08% before correction and it increased to 84.56% after correction. The increase in genome coverage shows that our method is able to correct Rmaps from across the genome. Furthermore it shows that even if Rmaps could not originally be reliably aligned to some regions of the genome, our method is sensitive enough to recover similar Rmaps from these regions and thus after correction the fraction of the genome covered by aligned Rmaps is higher. For the plum, the genome coverage dropped negligibly from 99.01% before error correction to 98.85% after error correction(which is less than 1% of the genome size).

In addition, as shown in Table 5, the running time and peak

memory usage was recorded for the plum and goat genome. Although these experiments have significant running times, (7.4 and 105.7 CPU days for plum and goat, respectively) these figures are not prohibitive given that this computation can easily be parallelized since the error correction process for each Rmap is independent. For example, we ran the goat genome on 20 machines and thus, it required a total of 126.84 hours for all Rmaps to be corrected. In addition, we note that error correction of a dataset will likely only be done once for any dataset so 5.2 human days for a large genome is not unreasonable. Lastly, the peak memory usage was 12.20 GB and 113.56 GB, for plum and goat, respectively, and thus, cOMet is able to run on any modern server.

Next, we ran experiments on the human dataset. Again, since we do not possess the error free Rmaps corresponding to the raw Rmaps for this dataset, we follow a similar evaluation method as in the previous experiments. We performed our evaluation on a *in silico* digested human reference genome (GenBank assembly accession: GCA_000001405.15, Genome Reference Consortium Human Build 38) using BspQI, which was the restriction enzyme that was used for generating the Rmap data. cOMet improves the S-score of 74.78% of the RMaps. The average S-score improves from 85.96 before error correction to 88.65 after error correction.

Conclusion

Error correction of high-throughput sequencing data has become an imperative pre-processing step in genome assembly since 2008 when Chaisson and Pevzner showed the dramatic improvement it can have on the quality of the assembly [3, 8, 20]. For example, after error correction the contig N50 size of an assembly of *Rhodabacter sphaeroides* improved from 233 bp to 7,793 bp using the same assembler [20]. Due to this inarguable benefit on genome assembly, many methods have been developed for error correction of sequence reads, including BFC [11], Coral [19], EULER [17, 3] and Reptile [27]. Unfortunately, even though there has been a massive effort into error correction of sequence data, there currently does not exist a publicly released method for error correction of Rmap data—a method that would likely improve the quality of genome-wide optical map assemblies, and allow such assemblies to be computed with greater efficiency.

In this paper, we presented cOMet, an error correction method for Rmap data, and demonstrate that it corrects and improves the quality of a high percentage of Rmaps in both the simulated and real datasets. As previously discussed, Rmap data is subject to high error rates. In addition to insertion and deletion errors, they contain sizing errors which necessitates the use of dynamic programming algorithm for pairwise alignment, and subsequently, assembly. By correcting a significant number of errors in Rmap data, cOMet can make it possible to use faster alignment methods [15, 10, 13], and explore the development of more efficient Rmap assembly algorithms.

Availability of source code

The cOMet software is written in C++ and is publicly available under GNU General Public License at https://github.com/kingufl/cOMet

Availability of Supporting Data

The optical mapping data for plum and goat is publicly available and can be accessed from their respective manuscripts. The simulated data for *E.coli* is provided in the github repository along with the python scripts used to generate it.

Acknowledgements

KM, DW, MM and CB were funded by the National Science Foundation (1618814) and LS was funded by Academy of Finland (grants 284598 (CoECGR), 308030, and 314170).

References

1. Bradnam KR, et al. Assemblathon 2: Evaluating *de novo* methods of genome assembly in three vertebrate species. GigaScience 2013;2(1):1–31.
2. Chaisson MJ, Tesler G. Mapping single molecule sequencing reads using basic local alignment with successive refinement (BLASR): application and theory. BMC Bioinformatics 2012;p. 238.
3. Chaisson MJ, Brinza D, Pevzner PA. De novo fragment assembly with short mate-paired reads: Does the read length matter? Genome Res 2009;19(2):336–346.
4. Chamala S, et al. Assembly and Validation of the Genome of the Nonmodel Basal Angiosperm *Amborella*. Science 2013;342(6165):1516–1517.
5. Church DM, et al. Lineage-Specific Biology Revealed by a Finished Genome Assembly of the Mouse. PLoS Biology 2009;7(5):e1000112+.
6. Dong Y, et al. Sequencing and Automated Whole-Genome Optical Mapping of the Genome of a Domestic Goat. Nature Biotechnology 2013;31(2):136–141.
7. Dong Y, et al., Sequencing and automated whole-genome optical mapping of the genome of a domestic goat (*Capra hircus*). Nature Biotechnol.; 2013.
8. Ekblom R, Wolf JBW. A field guide to whole-genome sequencing, assembly and annotation. Evolutionary Applications 2014;7(9):1026–1042.
9. Ganapathy G, et al. *De novo* high-coverage sequencing and annotated assemblies of the budgerigar genome. GigaScience 2014;3:11.
10. Leung AKY, et al. OMBlast: alignment tool for optical mapping using a seed-and-extend approach. Bioinformatics 2016;p. btw620.
11. Li H. BFC: correcting Illumina sequencing errors. Bioinformatics 2015;31(17):2885.
12. Li M, et al. Towards a More Accurate Error Model for BioNano Optical Maps. In: ISBRA 2016;. p. 67–79.
13. Mendelowitz LM, et al. Maligner: a fast ordered restriction map aligner. Bioinformatics 2016;32(7):1016–1022.
14. Miclotte G, Plaisance S, Rombauts S, Van de Peer Y, Audenaert P, Fostier J. OMSim: a simulator for optical map data. Bioinformatics 2017;p. 2740–2742.
15. Muggli MD, Puglisi SJ, Boucher C. In: Efficient indexed alignment of contigs to optical maps; 2014. p. 68–81.
16. Muggli MD, Puglisi SJ, Ronen R, Boucher C. Misassembly detection using paired-end sequence reads and optical mapping data. Bioinformatics 2015;31(12):i80–i88.
17. Pevzner PA, Tang H, Waterman MS. An Eulerian Path Approach to DNA Fragment Assembly. Proceedings of the National Academy of Sciences 2001;98(17):9748–9753.
18. Reslewic S, et al. Whole-Genome Shotgun Optical Mapping of *Rhodospirillum Rubrum*. Appl Environ Microbiol 2005;71(9):5511–5522.
19. Salmela L, Schröder J. Correcting errors in short reads by multiple alignments. Bioinformatics 2011;27(11):1455–1461.
20. Salzberg SL, et al. GAGE: A critical evaluation of

genome assemblies and assembly algorithms. Genome Res 2012;22(3):557–567.
21. Schwartz DC, et al. Ordered Restriction Maps of *Saccharomyces Cerevisiae* Chromosomes Constructed by Optical Mapping. Science 1993;262:110–114.
22. Shi L, et al. Long-read sequencing and de novo assembly of a Chinese genome. Nature Communications 2016 Jun;.
23. Smith TF, Waterman MS. Identification of common molecular subsequences. J Mol Biol 1981;147(1):195 – 197.
24. Teague B, et al. High-Resolution Human Genome Structure by Single-Molecule Analysis. Proc Natl Acad Sci USA 2010;107(24):10848–10853.
25. Valouev A, Schwartz DC, Zhou S, Waterman MS. An algorithm for assembly of ordered restriction maps from single DNA molecules. Proc Natl Acad Sci USA 2006;103(43):15770–15775.
26. Valouev A, et al. Alignment of optical maps. J Comp Biol 2006;13(2):442–462.
27. Yang X, Dorman KS, Aluru S. Reptile: representative tiling for short read error correction. Bioinformatics 2010;26(20):2526.
28. Zhang Q, et al., Genomic data of the plum (Prunus mume). GigaScience Database; 2014.
29. Zhou S, et al. A Whole-Genome Shotgun Optical Map of *Yersinia pestis* Strain KIM. Appl Environ Microbiol 2002;68(12):6321–6331.
30. Zhou S, et al. Shotgun Optical Mapping of the Entire *Leishmania major* Friedlin Genome. Mol Biochem Parasitol 2004;138(1):97–106.
31. Zhou S, et al. Validation of Rice Genome Sequence by Optical Mapping. BMC Genomics 2007;8(1):278.
32. Zhou S, et al. A Single Molecule Scaffold for the Maize Genome. PLoS Genetics 2009 11;5:e1000711.
33. Zhou S, Herschleb J, Schwartz DC. A single molecule system for whole genome analysis. Perspectives in Bioanalysis 2007;2:265–300.

Supplementary material

In Figure 6 We show the distribution of lengths of Rmaps whose S-score increases after error correction. From the distribution we can tell that our method is able to error correct Rmaps of all sizes. We also show the distribution of fragment sizes from Rmaps whose score increases after error correction in Figure 7.

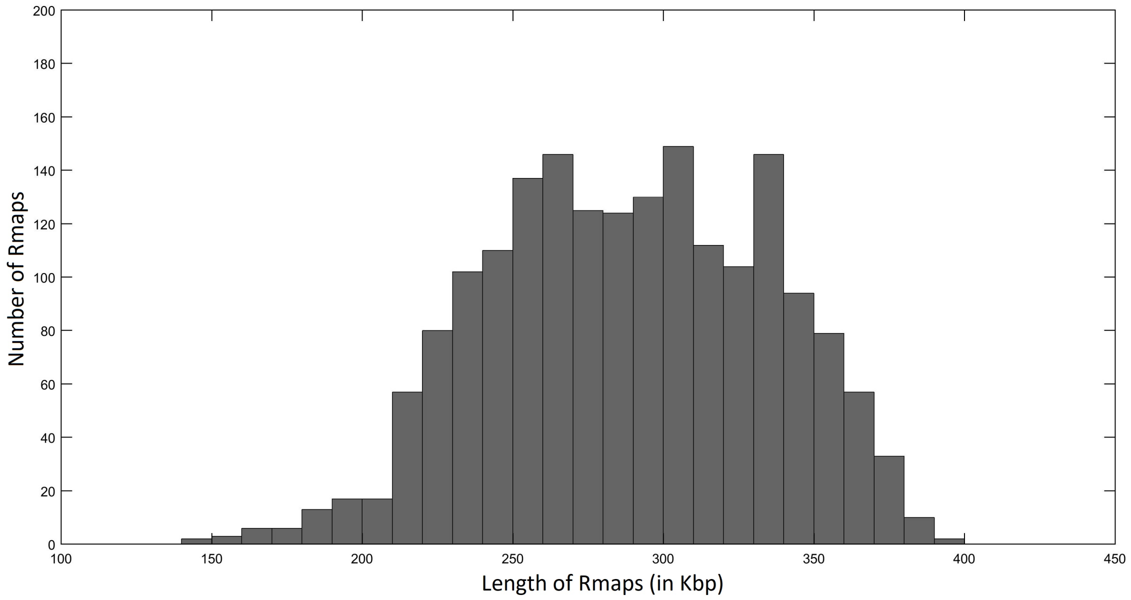

Figure 6. Distribution of Rmap lengths whose S-score increased after error correction. The Rmaps are simulated from the Ecoli K-12 substr. MG 1655 as explained in the text.

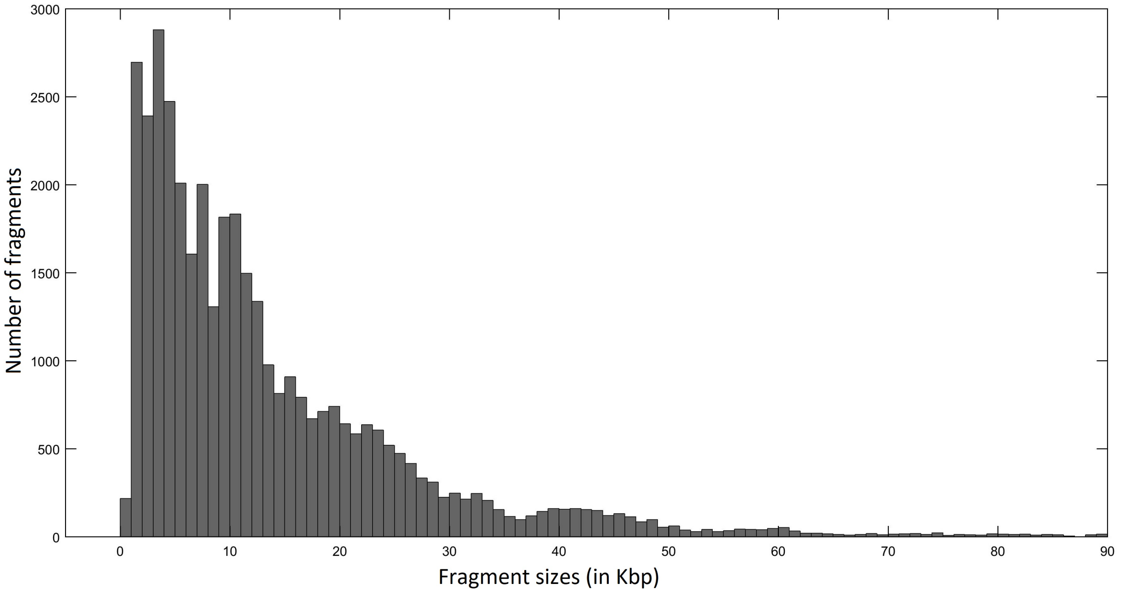

Figure 7. Distribution of fragment sizes of Rmaps whose S-score increased after error correction. The Rmaps are simulated from the Ecoli K-12 substr. MG 1655 as explained in the text.

logo file

Click here to download Figure gigascience-logo.eps

logo file

Click here to download Figure oup.eps

OXFORD

logo file

Click here to download Figure example-grid-100x100pt.png

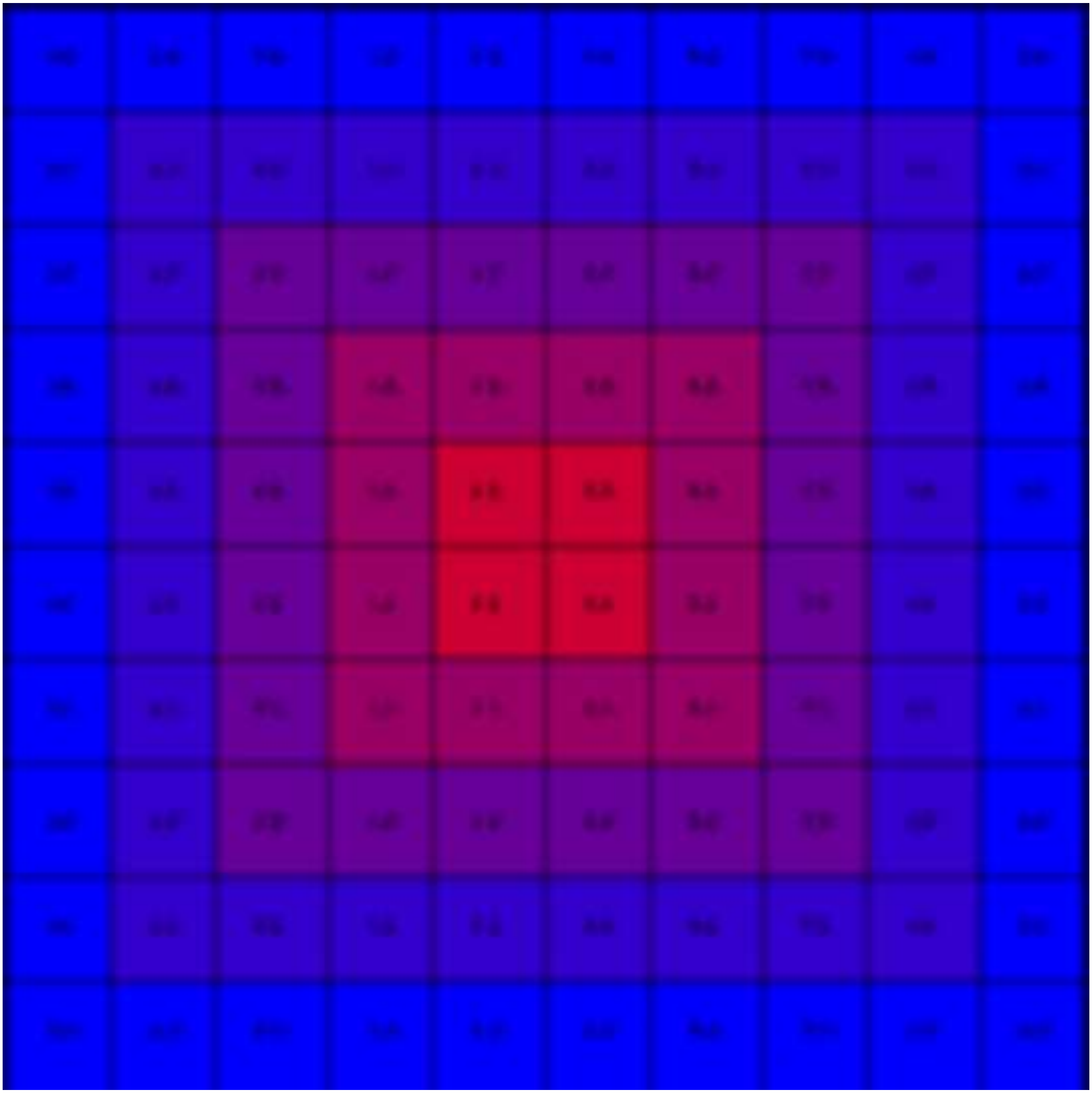

Reference pdf

Click here to access/download
Supplementary Material
error-correcting-optical.pdf

Response to reviewers Click here to download Personal Cover GigaScience_Response.pdf

March 6, 2018

RE: GIGA-D-17-00139R1
Error Correcting Optical Mapping Data
GigaScience
Kingshuk Mukherjee; Darshan Washimkar; Martin Muggli; Leena Salmela; Christina Boucher

Dear GigaScience Editorial Board:

We have revised our manuscript "Error Correcting Optical Mapping Data" (GIGA-D-17-00139R1) based on the assessment of both reviewers. Please see our response to their reports below.

Sincerely,
Kingshuk Mukherjee
PhD Candidate,
Department of Computer and Information Science and Engineering,
University of Florida
Gainesville, FL

Reviewer #1:

Dear Authors,

Thank you for addressing all the comments through this revision.

I only have one query regarding the new results added to the manuscript related to the assembly of corrected, uncorrected and error-free optical maps. In Table 2, it seems that the assembly of error-free optical maps have higher N50 than the assembly of error-corrected optical maps. Why is that the case? Some text highlighting why the error-corrected data performs better assembly than error-free data will be helpful.

Response: Thank you for your comment. The N50 is akin to the mean sequence length. In this case, the N50 is calculated for three numbers (error-free Rmaps) and two numbers (corrected Rmaps), respectively, and thus, not very informative due to the small sample size. Considering the number of assembled Rmaps and their lengths, we would argue that the assembly of the error-free and corrected Rmaps is fairly equal.

Reviewer #2:

The authors have addressed many of my original comments but I believe one major one did not get the attention it deserves. This is therefore a limited review that is focussed on just this issue as I believe it affects the generality of the method and its applicability across datasets.

This is the question of the simulated datasets used here and whether or not they are realistic in light of the stated goal to develop a general method that works with optical mapping data. The authors currently use one model that was developed for data from the BioNano system (even in this model, I don't see why the parameters cannot be varied to make the data harder to analyse). However, there are other models developed for other kinds of optical mapping data which have different parameters in their model for properties like the number of false cuts or missing cuts in a typical restriction map. The authors seem to find in their analysis that the real maps they use do not have properties similar to the simulated maps they work with. Without simulating maps that resemble real maps more, it would be hard to assess what the expected performance of the method would be on such real datasets. The authors correctly note that harder datasets would elicit worse performance. However, this is not the point. What we would want to see is that there is some level of robustness in the method and that results on harder datasets are not completely useless.

Response: Thank you for your comment. We have addressed this comment in the following manner:

1. *In addition to our simulated data, we simulated data using OMSim (Miclotte et al., Bioinformatics, 2017). When we began this work there was not a third-party optical map simulator and therefore, we had to write our own. We corrected this limitation in the latest version of our manuscript.*
2. *Using OMSim, we varied the parameters by several orders of magnitude above and below the default setting for OMSim. See the "Datasets" section for a complete discussion of the parameters and our variation from the default. Tables 6 and 7 show the performance of our method on these datasets. As you can see, our method still achieves a significant improvement in the error rate of the data.*
3. *Lastly, we added results for BioNano data generated from a human sample. Again, we demonstrate the utility of our method.*

We do want to note that current BioNano data is far less noisy in all regards than previous optical mapping datasets. This can be witnessed by considering the histograms of the S-scores. Hence, the Plum and Goat datasets are actually the hardest datasets from an assembly and error correction perspective.

UF | UNIVERSITY of FLORIDA

Herbert Wertheim College of Engineering
Department of Computer and Information Science and Engineering

300 Weil Hall
PO Box 116550
Gainesville, FL 32611-6550
352-392-6000
352-392-9673 Fax
christinaboucherl@ufl.edu

Dr. Laurie Goodman
Editor-in-Chief
GigaScience

May 30, 2017

Dear Dr. Goodman:

I am pleased to submit an original research article entitled "Error Correcting Optical Mapping Data" by Kingshuk Mukherjee, Darshan Washimker, Martin Muggli, Leena Salmela, and myself. This manuscript builds on our prior work on succinct data structures, error correction and optical mapping.

In this manuscript, we develop Comet, which is the first method proposed and released that error corrects Rmap data. Our experimental results demonstrate that it corrects 82.49% of insertion errors and 77.38% of deletion errors in Rmap data generated from the E. coli K-12 reference genome. It also successfully scales to large genomes, improving the quality of 78% and 99% of the Rmaps in the Plum and Goat genomes, respectively. We suggest Dr. David Schwartz as an editor. Further, we believe that this manuscript is appropriate for publication by GigaScience because of the algorithmic scope and focus on optical mapping, for which the suggested editor is a renowned expert.

This manuscript has not been published and is not under consideration for publication elsewhere. We have no conflicts of interest to disclose. In addition, if you feel that the manuscript is appropriate for your journal, we suggest the following reviewers:

Dr. Ali Bashir
Assistant Professor
Department of Genetics and Genomic Sciences
Icahn School of Medicine at Mount Sinai
EMAIL: ali.bashir@mssm.edu
PHONE: (212) 824-8949

Dr. Chan Ting Fung
Professor
School of Life Sciences
The Chinese University of Hong Kong
EMAIL: tf.chan@cuhk.edu.hk
PHONE (852) 3943 1216

Dr. Anton Valouev
Assistant Professor
Department of Preventive Medicine
Keck School of Medicine
University of Southern California
EMAIL: valouev@usc.edu
PHONE: (323) 442-7799

Thank you for your consideration

Sincerely,

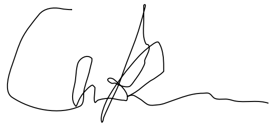

Christina Boucher

Assistant Professor
University of Florida
Department of Computer and Information Science and Engineering
