# Supplementary material for: Error correcting optical mapping data
Source: Gigascience. 2018 May 25;7(6):giy061. doi: 10.1093/gigascience/giy061 (PMC6007263; doi:10.1093/gigascience/giy061)
Supplement: Gigascience_OpticalMap_Review-Reviewer_1_Report_Original Submission_-Attachment.pdf [file giy061_gigascience_opticalmap_review-reviewer_1_report_original_submission_-attachment.pdf]

Dear Authors,

I have read your manuscript titled “Error Correcting Optical Mapping Data”, which discusses an algorithm cOMet to correct optical map data through alignment and consensus. Overall, the manuscript is well written, except at few places where the text is hard to follow. I think the method needs some more validation in terms of its effect on the downstream analysis such as optical map assembly.

Major Comments:

1. Page 2 line 6: In this paragraph, I think it will be a good idea to include a citation for the people who want to know more about why these kinds of errors take place.
2. Page 2 line 20: “This method is inherently computationally intensive” Can you please mention the algorithm complexity here?
3. Page 2 line 51 - 57: This description of the size quantization seems complicated. Although the idea is easy to follow, the way it is worded makes it harder to understand. I would recommend to make this description a bit simpler, maybe with an example or a figure.
4. Page 3 line 40: Is there any particular reason behind choosing a only three fragments (x, x+1, x+2)? How does this parameter affect the decision of saying that two optical maps are ‘related’? If you choose more fragments, will it be more specific but less sensitive?
5. Also, please provide overall complexity for your method.
6. Page 4 line 33: It is unclear how you correct the fragment size in R_i. For example in Figure 2, the sub-figure for the error correction, it is not clear how the corrected fragment sizes for R_i’ are computed based on the alignment grid shown in the same figure. I would recommend showing the fragment sizes in one of the columns of the alignment grid and show how would you compute the corrected fragment size using those values. This would make the text and the figure easier to follow.
7. Page 5 line 44: Can you show the distribution of fragment lengths for which the S-score increased after the correction? It will be interesting to see what is the typical fragment length that gets corrected with your correction method.
8. Page 6 line 31: “the genome coverage dropped negligibly…” This statement probably needs more discussion.
9. I ran the code for your method on the test data provided for ecoli. For the error free data it took 294 seconds and for the data with error it took 67.23 seconds. Why does it take more correction time for the error free data than the data with error?

Minor comments:

1. Citation style is different in abstract and the main text. Please make sure that the same style is followed throughout the manuscript.
2. Citation in the manuscript are not numbered in correct manner.

3. The source code repository has a bin folder with the binary file. Because of this, when I run make, nothing happens. Please provide a rule for ‘make clean’ in case someone wants to rebuild the code.
