# Supplementary material for: Error correcting optical mapping data
Source: Gigascience. 2018 May 25;7(6):giy061. doi: 10.1093/gigascience/giy061 (PMC6007263; doi:10.1093/gigascience/giy061)
Supplement: Response_to_Reviewer_Comments_Original_Submission.pdf [file giy061_response_to_reviewer_comments_original_submission.pdf]

Author's Response To Reviewer Comments

Close

GIGA-D-17-00139
Error Correcting Optical Mapping Data
GigaScience

Dear GigaScience Editors,

We are submitting a revised manuscript that addresses the reviewers' comments. See our response to these comments below.

Best,
Kingshuk Mukherjee

Reviewer reports:

Reviewer #1, Jay Ghurye: Dear Authors,

I have read your manuscript titled "Error Correcting Optical Mapping Data", which discusses an algorithm cOMet to correct optical map data through alignment and consensus. Overall, the manuscript is well written, except at few places where the text is hard to follow. I think the method needs some more validation in terms of its effect on the downstream analysis such as optical map assembly.

Major Comments:

Page 2 line 6: In this paragraph, I think it will be a good idea to include a citation for the people who want to know more about why these kinds of errors take place.

Thank you for this comment. We have added the following two citations to address this comment:
Li M, et al. Towards a More Accurate Error Model for BioNano Optical Maps. In: ISBRA 2016. p. 67–79.
Valouev A, et al. Alignment of optical maps. J Comp Biol 2006;13(2):442–462.

Page 2 line 20: "This method is inherently computationally intensive" Can you please mention the algorithm complexity here?

Thank you for this comment. We have added a couple sentences about the complexity.

Page 2 line 51 - 57: This description of the size quantization seems complicated. Although the idea is easy to follow, the way it is worded makes it harder to understand. I would recommend to make this description a bit simpler, maybe with an example or a figure.

We have altered the description and added an example to make the quantization easier to follow.

Page 3 line 40: Is there any particular reason behind choosing only three fragments (x, x+1, x+2)?

How does this parameter affect the decision of saying that two optical maps are 'related'? If you choose more fragments, will it be more specific but less sensitive?
Thank you for this comment. This is just a simple heuristic and no, there is no specific reason that three is used rather than 4. We have made it clear in the paper that this is a simple heuristic only to reduce the number of pairs of Rmaps to which the full alignment method needs to be ran.

Also, please provide overall complexity for your method.

We have added a section about the complexity.

Page 4 line 33: It is unclear how you correct the fragment size in R_i. For example in Figure 2, the sub-figure for the error correction, it is not clear how the corrected fragment sizes for R_i' are computed based on the alignment grid shown in the same figure. I would recommend showing the fragment sizes in one of the columns of the alignment grid and show how would you compute the corrected fragment size using those values. This would make the text and the figure easier to follow.

We have addressed this by showing the aligned fragment sizes for three fragments of R_i. We have used the third, fourth and fifth fragments of R_i in Figure 2 to illustrate the error correction.

Page 5 line 44: Can you show the distribution of fragment lengths for which the S-score increased after the correction? It will be interesting to see what is the typical fragment length that gets corrected with your correction method.

I think what you mean is the length of the Rmaps not the length of the fragments. Thus, we give an histogram for the lengths of the Rmaps for which the S-score increased. See Figures 6 and 7 in the Supplementary Material.

Page 6 line 31: "the genome coverage dropped negligibly..." This statement probably needs more discussion.

We have added a sentence clarifying that negligible in this case means less than 1% of the total genome size.

I ran the code for your method on the test data provided for ecoli. For the error free data it took 294 seconds and for the data with error it took 67.23 seconds. Why does it take more correction time for the error free data than the data with error?

The same thing happens with PacBio error correction methods. The reason is with the error free data, less number of Rmaps are filtered out by the filtering heuristic. Therefore the DP aligner performs many more pairwise alignments (which is the bottleneck in the error correction).

Minor comments:
Citation style is different in abstract and the main text. Please make sure that the same style is followed throughout the manuscript.

This was done purposefully because in NCBI website, etc. the abstract is available but the full paper has to be explicitly downloaded. Therefore the citation numbers lose context. Nonetheless, we changed it.

Citation in the manuscript are not numbered in correct manner.
This is the latex template for the journal. The journal uses alphabetical ordering.

The source code repository has a bin folder with the binary file. Because of this, when I run make, nothing happens. Please provide a rule for 'make clean' in case someone wants to rebuild the code.

“make clean” has been added.

Reviewer #2, N. Nagarajan:

Abstract:

1) Page 1, Line 32: I believe this sentence can be rephrased as the attempt at distinguishing Rmap and optical mapping data here is not clear. Also, it should be "challenges ... are".

We agree. This was a very good comment. We have fixed this.

2) Page 1, Line 33-34: Perhaps rephrase "data is integral" because it is possible to misinterpret this sentence.

We also agree. This was a very good comment. We have fixed this.

3) Page 1, Line 35-38: The sensitivity of the method is highlighted without any reference to its precision. I believe many readers would wonder about how specific cOMet's corrections are.

Thank you. This is a nice comment. We have added a sentence about the precision. This actually strengthens the paper because our precision is very high. On page 6 in “Experiments with Simulated Data”: “Our method also has high precision. Out of the deletion errors corrected, 98.25% are true errors. Similarly, out of the insertion errors corrected, 82.19% are true.”

3) Page 1, Line 37: " , " -> ", ".

We have fixed this.

Introduction:

4) "camera.Computer" -> "camera. Computer"

We have fixed this.

5) What are "fragment size substitution errors"?

We have fixed this.

6) Page 2, line 7: This statement needs to be qualified as it is indeed possible to use Rmap data without assembly.

That is a valid point. We have removed this sentence.

Background:

7) Page 2, line 48: Note sure what the reference to cells is about. Are these biological cells or mapping cells?

We have "cells of the organism", which we believe implies biological cells.

8) Page 2: "which depends on actual length of the fragment. For example [20]". The reference seems to be oddly placed and the previous sentence seems to be missing references.

We have fixed this.

9) "Smith and Waterman alignment" -> "Smith-Waterman alignment"

We have fixed this.

Methods:

10) It will be nice to have default values for each parameter listed here as this will help the reader understand how the method might behave.

We have the default parameters in "Experiments and Discussion" on page 5, last sentence of the first paragraph. We have also added these to "Method"; at the end of each subsection there is the default parameter and a short explanation to help the reader understand how the method behaves as the parameters are altered.

11) This sentence is not quite clear to me: "Next, we consider all combinations of matching the fragments at positions ...". If x is matched to y and x+1 to y+1, how is that compared to a matching where x,y is matched to x+1,y+1. In the first case there are two values for "difference in the total size" and in the second case there is only one value. Its possible that I don't understand this correctly but perhaps there is a better way of presenting this as well.

We have given a mathematical formulation of our evaluation criteria at the top of page 3. To answer the question, for each combination, we consider the total size difference. That is, in the first case we will consider the size difference between R_i[x] and R_j[y] and in the second case we consider the size difference between R_i[x]+R_i[x+1] and R_j[y]+R_j[y+1].

12) How was the S-score threshold of 8 derived?

Thank you. That is a very nice insight. We have added a histogram showing the distribution of S-scores for related Rmaps vs unrelated Rmaps on page 5. Setting the S-score to 8 includes 95.96% of related Rmaps. Therefore we chose this parameter setting.

Datasets:

13) This needs greater detail: " appropriately parametrized Gaussian distribution ...".

We have added a more detailed description of the error model which we follow for simulating the optical maps in the Background section. Specially, this is found on page 2, second column, second

paragraph.

14) Are the parameters used for the simulations here inferred on a genome-wide basis or on a per map basis? Which datasets were these estimated based on? There is likely to be variability in these parameters based on runs, genomes and restriction enzyme. How does cOMet perform on harder datasets (see e.g. Verzotto et al. 2016)?

Thank you for this point. It is a bit unclear what is meant by “harder datasets” as the data is simulated based on a mathematical model of the error that applies to all data of this nature. For example, with Illumina and/or PacBio error correction a single model of the error rate is used (not multiple). You can of course change the error model but it is unclear to me as to why you would want to. If you increase the error rate the performance of the error correction (of all error correction methods) will degrade but the point is to use a mathematical model of the data that has been peer-reviewed. To date, there has only been one such model, which is the one we use in this paper.

15) The number of errors in the simulated datasets seems to be lower than what you would expect (~10 insertion errors per genome copy i.e. ~3000 errors in 300 genome copies). What explains this discrepancy? The S-scores for the simulated dataset are also much larger than for real datasets. Is it because the simulation represents too easy a dataset?

This is a very good observation. If the insertion errors were added to the whole genome optical map then we can expect ~10 errors per genome copy. However, the errors were added to each simulated Rmap individually. Since the Rmaps have an average length of 280 kbp, and the insertion errors are added with a probability of x errors per unit length(unit length being 400kbp), therefore the random variable used in the simulation caused many Rmaps to not get any insertion error. This leads to the observed discrepancy.

We followed the error model of Li et al for generating the simulated datasets. It is possible the real datasets have different distribution of errors (as they were built on a different platform- OpGen). It is also possible to have low alignment scores because of the fragmented nature of the draft genomes and possible misassemblies present in the genome of the real datasets.

Experiments:

16) Is the FPR calculation correct? I get very different denominators for insertion and deletion errors and from what I understand, that shouldn't be the case. Also, has cOMet been essentially optimised for this dataset (choice of parameters)? How would it do on an entirely different simulated dataset? How robust are the parameter choices? I believe experiments with more realistic datasets are needed to establish cOMet's performance level. Also, if we take the consensus alignment and assembly for OM data as a standard of truth then there should be many real datasets where cOMet's performance can be rigorously evaluated.

The FPR calculation is correct. The denominators (number of fragments not having insertion/deletion) errors is very high compared to the numerator (number of fragments falsely assessed to have insertion/deletion errors). Hence we get low FPR. The denominator values for insertion and deletion errors have a difference of 6,931 which is the difference in the number of insertion and deletion errors present in the simulated data. For example, there are 48,188 number of fragments across all Rmaps and there are 7,485 deletion errors. Therefore 40,703 fragments do not have deletion errors. Number of fragments falsely assessed to have deletion errors is 102. Therefore we arrive at FPR value 102/40,703 = 0.25% for the deletion errors. Similarly the FPR value for insertion errors is 99/47,634 = 0.21% for the insertion errors.

We simulated our dataset based on the error model from Li et al. It is possible that with a different error model, the optimum setting of the parameters will be slightly different. We have added discussion about how each parameter affects the performance. This will guide an user how to vary the parameters while working with a different error model.

17) Page 5: How were the alignments to reference computed? Why are the uncorrected Rmaps not allowed to align to a different best position to compute their S-score?

Thank you for this comment. The alignment to the reference was computed by choosing the position where the error corrected Rmaps aligned with the highest score. We acknowledge in the paper that this process is error prone because of the fragmented nature of the draft genomes and possible mis-assemblies present in the genome. For the real dataset, it is not clear where the true alignment of an Rmap lies, and therefore we chose to use this method. To demonstrate that our method is able to correct Rmaps across the genome, we have also shown that the genome coverage of the alignments after error correction is good. This shows that the method does not have significant bias towards some region of the genome.

18) The real datasets seem to uncover a different relative frequency of insertion and deletion errors than the simulated datasets where the proportion is 1:10. What explains this difference? If you assume that all of them are true errors how does the distribution along Rmaps look compared to the parameters used for the simulation?

This is a very good comment. It is true that the real datasets seem to uncover a different relative frequency of insertion and deletion errors compared to what we got from the simulated data. There are two possible explanations. One, is that the real data comes from a different platform (the OpGen platform) and therefore, possibly has a different distribution of the errors. Two, the errors reported are the errors that could be discovered and corrected using our method. The true distribution of errors could be different. As this data is still in transition in its development the error rate will fluctuate from previous datasets (that were perhaps generated only a couple years ago) to current datasets. The error rate is also more susceptible to fluctuation due to laboratory (NGS) data. This is just one of the challenges that arise from working with this data. We feel the most scientifically appropriate way to deal with this challenge is to generate the data according to a peer-reviewed, published error model.

19) It seems that the primary use for error correction as advocated by the authors is for accelerating map assembly. Is the error correction provided by cOMet sufficient to enable faster assembly using existing tools? Showing results for this is critical to establishing cOMet's utility to the community.

To demonstrate the usefulness of error correction, we conducted additional experiments. We assembled the Rmaps before and after error correction using the Valouev assembler and found that the error corrected Rmaps could be assembled into more contiguous contigs with higher N50 value than the uncorrected data. We also aligned the assembled contigs to the genome-wide reference to located the positions of the contigs on the reference. The results are summarized in Table 2.

20) Page 6: "only be done for once" -> "only be done once"

We have fixed this.

21) Will additional iterations of applying cOMet reduce errors further?

This is a good comment. We did try this and it did not improve the error rate. This is similar to short read / PacBio error correction methods. Re-iteration also doesn’t improve the quality of the data. Moreover, since these methods are heuristics, there is no way to prove or predict their behavior.

Conclusion:

22) Page 6, line 58: "cpuntless" -> "many"

We have fixed this.

Close
