# Supplementary material for: Error correcting optical mapping data
Source: Gigascience. 2018 May 25;7(6):giy061. doi: 10.1093/gigascience/giy061 (PMC6007263; doi:10.1093/gigascience/giy061)
Supplement: Response_to_Reviewer_Comments_Revision_1.pdf [file giy061_response_to_reviewer_comments_revision_1.pdf]

Author's Response To Reviewer Comments

Close

March 6, 2018

RE: GIGA-D-17-00139R1
Error Correcting Optical Mapping Data
GigaScience
Kingshuk Mukherjee; Darshan Washimkar; Martin Muggli; Leena Salmela; Christina Boucher

Dear GigaScience Editorial Board:

We have revised our manuscript "Error Correcting Optical Mapping Data" (GIGA-D-17-00139R1) based on the assessment of both reviewers. Please see our response to their reports below.

Sincerely,
Kingshuk Mukherjee
PhD Candidate,
Department of Computer and Information Science and Engineering,
University of Florida
Gainesville, FL

-
Reviewer #1:

Dear Authors,

Thank you for addressing all the comments through this revision.

I only have one query regarding the new results added to the manuscript related to the assembly of corrected, uncorrected and error-free optical maps. In Table 2, it seems that the assembly of error-free optical maps have higher N50 than the assembly of error-corrected optical maps. Why is that the case? Some text highlighting why the error-corrected data performs better assembly than error-free data will be helpful.

Response: Thank you for your comment. The N50 is akin to the mean sequence length. In this case, the N50 is calculated for three numbers (error-free Rmaps) and two numbers (corrected Rmaps), respectively, and thus, not very informative due to the small sample size. Considering the number of assembled Rmaps and their lengths, we would argue that the assembly of the error-free and corrected Rmaps is fairly equal.

-

Reviewer #2:

The authors have addressed many of my original comments but I believe one major one did not get

the attention it deserves. This is therefore a limited review that is focussed on just this issue as I believe it affects the generality of the method and its applicability across datasets.

This is the question of the simulated datasets used here and whether or not they are realistic in light of the stated goal to develop a general method that works with optical mapping data. The authors currently use one model that was developed for data from the BioNano system (even in this model, I don't see why the parameters cannot be varied to make the data harder to analyse). However, there are other models developed for other kinds of optical mapping data which have different parameters in their model for properties like the number of false cuts or missing cuts in a typical restriction map. The authors seem to find in their analysis that the real maps they use do not have properties similar to the simulated maps they work with. Without simulating maps that resemble real maps more, it would be hard to assess what the expected performance of the method would be on such real datasets. The authors correctly note that harder datasets would elicit worse performance. However, this is not the point. What we would want to see is that there is some level of robustness in the method and that results on harder datasets are not completely useless.

Response: Thank you for your comment. We have addressed this comment in the following manner:

In addition to our simulated data, we simulated data using OMSim (Miclotte et al., Bioinformatics, 2017). When we began this work there was not a third-party optical map simulator and therefore, we had to write our own. We corrected this limitation in the latest version of our manuscript.

Using OMSim, we varied the parameters by several orders of magnitude above and below the default setting for OMSim. See the "Datasets" section for a complete discussion of the parameters and our variation from the default. Tables 6 and 7 show the performance of our method on these datasets. As you can see, our method still achieves a significant improvement in the error rate of the data.

Lastly, we added results for BioNano data generated from a human sample. Again, we demonstrate the utility of our method.

We do want to note that current BioNano data is far less noisy in all regards than previous optical mapping datasets. This can be witnessed by considering the histograms of the S-scores. Hence, the Plum and Goat datasets are actually the hardest datasets from an assembly and error correction perspective.

Close
