# Supplementary material for: Error correcting optical mapping data
Source: Gigascience. 2018 May 25;7(6):giy061. doi: 10.1093/gigascience/giy061 (PMC6007263; doi:10.1093/gigascience/giy061)
Supplement: Response_to_Reviewer_Comments_Revision_2.pdf [file giy061_response_to_reviewer_comments_revision_2.pdf]

Author's Response To Reviewer Comments

Close

1) Data citation:

I have cited the plum, goat, human and my the supporting data for this paper. However, the DOIs donot appear on the manuscript even though they are present in the bibtex file.

2) Section "Availability of source code":

I have done this.

3) RRID:

I have included the RRID.

4) Source files:

I have submitted all source files as well as the reference pdf through the submission portal.

5) Archival copy in GigaDB:

I have cited the availability of supporting data from the URL provided to me. It appears as citation no 17 in the paper. However as mentioned in point 1, I cannot make the DOI appear.

Please let me know if I need to do anything else.

Close
