# Supplementary material for: Error correcting optical mapping data
Source: Gigascience. 2018 May 25;7(6):giy061. doi: 10.1093/gigascience/giy061 (PMC6007263; doi:10.1093/gigascience/giy061)
Supplement: Reviewer_1_Report_(Original_Submission) -- Jay Ghurye — 7/21/2017 Reviewed [file giy061_reviewer_1_report_(original_submission).pdf]

Reviewer Report

Title: Error Correcting Optical Mapping Data

Version: Original Submission **Date:** 7/21/2017

Reviewer name: Jay Ghurye

Reviewer Comments to Author:

Dear Authors,I have read your manuscript titled "Error Correcting Optical Mapping Data", which discusses an algorithm cOMet to correct optical map data through alignment and consensus. Overall, the manuscript is well written, except at few places where the text is hard to follow. I think the method needs some more validation in terms of its effect on the downstream analysis such as optical map assembly. Major Comments:Page 2 line 6: In this paragraph, I think it will be a good idea to include a citation for the people who want to know more about why these kinds of errors take place. Page 2 line 20: "This method is inherently computationally intensive" Can you please mention the algorithm complexity here? Page 2 line 51 - 57: This description of the size quantization seems complicated. Although the idea is easy to follow, the way it is worded makes it harder to understand. I would recommend to make this description a bit simpler, maybe with an example or a figure.Page 3 line 40: Is there any particular reason behind choosing only three fragments (x, x+1, x+2)? How does this parameter affect the decision of saying that two optical maps are 'related'? If you choose more fragments, will it be more specific but less sensitive?Also, please provide overall complexity for your method. Page 4 line 33: It is unclear how you correct the fragment size in R_i. For example in Figure 2, the sub-figure for the error correction, it is not clear how the corrected fragment sizes for R_i' are computed based on the alignment grid shown in the same figure. I would recommend showing the fragment sizes in one of the columns of the alignment grid and show how would you compute the corrected fragment size using those values. This would make the text and the figure easier to follow. Page 5 line 44: Can you show the distribution of fragment lengths for which the S-score increased after the correction? It will be interesting to see what is the typical fragment length that gets corrected with your correction method. Page 6 line 31: "the genome coverage dropped negligibly..." This statement probably needs more discussion. I ran the code for your method on the test data provided for ecoli. For the error free data it took 294 seconds and for the data with error it took 67.23 seconds. Why does it take more correction time for the error free data than the data with error?Minor comments:Citation style is different in abstract and the main text. Please make sure that the same style is followed throughout the manuscript. Citation in the manuscript are not numbered in correct manner. The source code repository has a bin folder with the binary file. Because of this, when I run make, nothing happens. Please provide a rule for 'make clean' in case someone wants to rebuild the code.

Level of Interest

Please indicate how interesting you found the manuscript: Choose an item.

Quality of Written English

Please indicate the quality of language in the manuscript: Choose an item.

Declaration of Competing Interests

Please complete a declaration of competing interests, considering the following questions:

- Have you in the past five years received reimbursements, fees, funding, or salary from an organisation that may in any way gain or lose financially from the publication of this manuscript, either now or in the future?
- Do you hold any stocks or shares in an organisation that may in any way gain or lose financially from the publication of this manuscript, either now or in the future?
- Do you hold or are you currently applying for any patents relating to the content of the manuscript?
- Have you received reimbursements, fees, funding, or salary from an organization that holds or has applied for patents relating to the content of the manuscript?
- Do you have any other financial competing interests?
- Do you have any non-financial competing interests in relation to this paper?

If you can answer no to all of the above, write 'I declare that I have no competing interests' below. If your reply is yes to any, please give details below.

I declare that I have no competing interests

I agree to the open peer review policy of the journal. I understand that my name will be included on my report to the authors and, if the manuscript is accepted for publication, my named report including any attachments I upload will be posted on the website along with the authors' responses. I agree for my report to be made available under an Open Access Creative Commons CC-BY license (http://creativecommons.org/licenses/by/4.0/). I understand that any comments which I do not wish to be included in my named report can be included as confidential comments to the editors, which will not be published.

I agree to the open peer review policy of the journal

To further support our reviewers, we have joined with Publons, where you can gain additional credit to further highlight your hard work (see: https://publons.com/journal/530/gigascience). On publication of this paper, your review will be automatically added to Publons, you can then choose whether or not to claim your Publons credit. I understand this statement.

Yes
