# Supplementary material for: Error correcting optical mapping data
Source: Gigascience. 2018 May 25;7(6):giy061. doi: 10.1093/gigascience/giy061 (PMC6007263; doi:10.1093/gigascience/giy061)
Supplement: Reviewer_2_Report_(Original_Submission) -- Niranjan Nagarajan, PhD — 9/6/2017 Reviewed [file giy061_reviewer_2_report_(original_submission).pdf]

Reviewer Report

Title: **Error Correcting Optical Mapping Data**

Version: **Original Submission** **Date:** 9/6/2017

Reviewer name: **Niranjan Nagarajan, PhD**

Reviewer Comments to Author:

Abstract:1) Page 1, Line 32: I believe this sentence can be rephrased as the attempt at distinguishing Rmap and optical mapping data here is not clear. Also, it should be "challenges ... are".2) Page 1, Line 33-34: Perhaps rephrase "data is integral" because it is possible to misinterpret this sentence.3) Page 1, Line 35-38: The sensitivity of the method is highlighted without any reference to its precision. I believe many readers would wonder about how specific cOMet's corrections are.3) Page 1, Line 37: " , " -> ", ".Introduction:4) "camera.Computer" -> "camera. Computer"5) What are "fragment size substitution errors"?6) Page 2, line 7: This statement needs to be qualified as it is indeed possible to use Rmap data without assembly.Background:7) Page 2, line 48: Note sure what the reference to cells is about. Are these biological cells or mapping cells?8) Page 2: "which depends on actual length of the fragment. For example [20]". The reference seems to be oddly placed and the previous sentence seems to be missing references.9) "Smith and Waterman alignment" -> "Smith-Waterman alignment"Methods:10) It will be nice to have default values for each parameter listed here as this will help the reader understand how the method might behave.11) This sentence is not quite clear to me: "Next, we consider all combinations of matching the fragments at positions ...". If x is matched to y and x+1 to y+1, how is that compared to a matching where x,y is matched to x+1,y+1. In the first case there are two values for "difference in the total size" and in the second case there is only one value. Its possible that I don't understand this correctly but perhaps there is a better way of presenting this as well.12) How was the S-score threshold of 8 derived?Datasets:13) This needs greater detail: " appropriately parametrized Gaussian distribution ...".14) Are the parameters used for the simulations here inferred on a genome-wide basis or on a per map basis? Which datasets were these estimated based on? There is likely to be variability in these parameters based on runs, genomes and restriction enzyme. How does cOMet perform on harder datasets (see e.g. Verzotto et al. 2016)?15) The number of errors in the simulated datasets seems to be lower than what you would expect (~10 insertion errors per genome copy i.e. ~3000 errors in 300 genome copies). What explains this discrepancy? The S-scores for the simulated dataset are also much larger than for real datasets. Is it because the simulation represents too easy a dataset?Experiments:16) Is the FPR calculation correct? I get very different denominators for insertion and deletion errors and from what I understand, that shouldn't be the case. Also, has cOMet been essentially optimised for this dataset (choice of parameters)? How would it do on an entirely different simulated dataset? How robust are the parameter choices? I believe experiments with more realistic datasets are needed to establish cOMet's performance level. Also, if we take the consensus alignment and assembly for OM data as a standard of truth then there should be many real datasets where cOMet's performance can be rigorously evaluated.17) Page 5: How were the alignments to reference computed? Why are the uncorrected Rmaps not allowed to align to a different best position to compute their S-score?18) The real datasets seem to uncover a different relative frequency of insertion and deletion errors than the simulated datasets where the proportion is 1:10. What explains this difference? If you assume that all of them are true errors how does the distribution along Rmaps look compared to the parameters used for the simulation?19) It seems that the primary use for error correction as advocated by the authors is for accelerating map assembly. Is the error correction provided by cOMet sufficient to enable faster assembly using existing tools? Showing results for this is critical to establishing cOMet's utility to the community.20) Page 6: "only be done for once" -> "only be done once"21) Will additional iterations of applying cOMet reduce errors further?Conclusion: 22) Page 6, line 58: "cpuntless" -> "many"

Level of Interest

Please indicate how interesting you found the manuscript: An article whose findings are important to those with closely related research interests

Quality of Written English

Please indicate the quality of language in the manuscript: Needs some language corrections before being published

Declaration of Competing Interests

Please complete a declaration of competing interests, considering the following questions:

- Have you in the past five years received reimbursements, fees, funding, or salary from an organisation that may in any way gain or lose financially from the publication of this manuscript, either now or in the future?
- Do you hold any stocks or shares in an organisation that may in any way gain or lose financially from the publication of this manuscript, either now or in the future?
- Do you hold or are you currently applying for any patents relating to the content of the manuscript?
- Have you received reimbursements, fees, funding, or salary from an organization that holds or has applied for patents relating to the content of the manuscript?
- Do you have any other financial competing interests?
- Do you have any non-financial competing interests in relation to this paper?

If you can answer no to all of the above, write 'I declare that I have no competing interests' below. If your reply is yes to any, please give details below.

Yes - we have applied for patents related to our earlier work on optical mapping data analysis (https://gigascience.biomedcentral.com/articles/10.1186/s13742-016-0110-0). This work is however quite different from the manuscript being reviewed.

I agree to the open peer review policy of the journal. I understand that my name will be included on my report to the authors and, if the manuscript is accepted for publication, my named report including any attachments I upload will be posted on the website along with the authors' responses. I agree for my report to be made available under an Open Access Creative Commons CC-BY license (http://creativecommons.org/licenses/by/4.0/). I understand that any comments which I do not wish to be included in my named report can be included as confidential comments to the editors, which will not be published.

I agree to the open peer review policy of the journal

To further support our reviewers, we have joined with Publons, where you can gain additional credit to further highlight your hard work (see: https://publons.com/journal/530/gigascience). On publication of this paper, your review will be automatically added to Publons, you can then choose whether or not to claim your Publons credit. I understand this statement.

Yes
