# Supplementary material for: Error correcting optical mapping data
Source: Gigascience. 2018 May 25;7(6):giy061. doi: 10.1093/gigascience/giy061 (PMC6007263; doi:10.1093/gigascience/giy061)
Supplement: Reviewer_2_Report_(Revision_1) - Niranjan Nagarajan, PhD — 1/28/2018 Reviewed [file giy061_reviewer_2_report_(revision_1).pdf]

Reviewer Report

Title: Error Correcting Optical Mapping Data

Version: Revision 1 Date: 1/28/2018

Reviewer name: Niranjan Nagarajan, PhD

Reviewer Comments to Author:

The authors have addressed many of my original comments but I believe one major one did not get the attention it deserves. This is therefore a limited review that is focussed on just this issue as I believe it affects the generality of the method and its applicability across datasets. This is the question of the simulated datasets used here and whether or not they are realistic in light of the stated goal to develop a general method that works with optical mapping data. The authors currently use one model that was developed for data from the BioNano system (even in this model, I don't see why the parameters cannot be varied to make the data harder to analyse). However, there are other models developed for other kinds of optical mapping data which have different parameters in their model for properties like the number of false cuts or missing cuts in a typical restriction map. The authors seem to find in their analysis that the real maps they use do not have properties similar to the simulated maps they work with. Without simulating maps that resemble real maps more, it would be hard to assess what the expected performance of the method would be on such real datasets. The authors correctly note that harder datasets would elicit worse performance. However, this is not the point. What we would want to see is that there is some level of robustness in the method and that results on harder datasets are not completely useless.

Level of Interest

Please indicate how interesting you found the manuscript: An article whose findings are important to those with closely related research interests

Quality of Written English

Please indicate the quality of language in the manuscript: Acceptable

Declaration of Competing Interests

Please complete a declaration of competing interests, considering the following questions:

- Have you in the past five years received reimbursements, fees, funding, or salary from an organisation that may in any way gain or lose financially from the publication of this manuscript, either now or in the future?
- Do you hold any stocks or shares in an organisation that may in any way gain or lose financially from the publication of this manuscript, either now or in the future?
- Do you hold or are you currently applying for any patents relating to the content of the manuscript?

- Have you received reimbursements, fees, funding, or salary from an organization that holds or has applied for patents relating to the content of the manuscript?
- Do you have any other financial competing interests?
- Do you have any non-financial competing interests in relation to this paper?

If you can answer no to all of the above, write 'I declare that I have no competing interests' below. If your reply is yes to any, please give details below.

Same as in the case of the original review

I agree to the open peer review policy of the journal. I understand that my name will be included on my report to the authors and, if the manuscript is accepted for publication, my named report including any attachments I upload will be posted on the website along with the authors' responses. I agree for my report to be made available under an Open Access Creative Commons CC-BY license (http://creativecommons.org/licenses/by/4.0/). I understand that any comments which I do not wish to be included in my named report can be included as confidential comments to the editors, which will not be published.

I agree to the open peer review policy of the journal

To further support our reviewers, we have joined with Publons, where you can gain additional credit to further highlight your hard work (see: https://publons.com/journal/530/gigascience). On publication of this paper, your review will be automatically added to Publons, you can then choose whether or not to claim your Publons credit. I understand this statement.

Yes
